# miR-275/305 cluster is essential for maintaining energy metabolic homeostasis by the insulin signaling pathway in *Bactrocera dorsalis*

**DOI:** 10.1371/journal.pgen.1010418

**Published:** 2022-10-05

**Authors:** Junfei Xie, Hao Chen, Wenping Zheng, Zhaohui Cai, Xiaoxue Li, Hongyu Zhang

**Affiliations:** Key Laboratory of Horticultural Plant Biology (Ministry of Education), Hubei Hongshan Laboratory, Institute of Urban and Horticultural Entomology, College of Plant Science and Technology, Huazhong Agricultural University, Wuhan, People’s Republic of China; University of Helsinki: Helsingin Yliopisto, FINLAND

## Abstract

Increasing evidence indicates that miRNAs play crucial regulatory roles in various physiological processes of insects, including systemic metabolism. However, the molecular mechanisms of how specific miRNAs regulate energy metabolic homeostasis remain largely unknown. In the present study, we found that an evolutionarily conserved miR-275/305 cluster was essential for maintaining energy metabolic homeostasis in response to dietary yeast stimulation in *Bactrocera dorsalis*. Depletion of miR-275 and miR-305 by the CRISPR/Cas9 system significantly reduced triglyceride and glycogen contents, elevated total sugar levels, and impaired flight capacity. Combined *in vivo* and *in vitro* experiments, we demonstrated that miR-275 and miR-305 can bind to the 3’UTR regions of *SLC2A1* and *GLIS2* to repress their expression, respectively. RNAi-mediated knockdown of these two genes partially rescued metabolic phenotypes caused by inhibiting miR-275 and miR-305. Furthermore, we further illustrated that the miR-275/305 cluster acting as a regulator of the metabolic axis was controlled by the insulin signaling pathway. In conclusion, our work combined genetic and physiological approaches to clarify the molecular mechanism of metabolic homeostasis in response to different dietary stimulations and provided a reference for deciphering the potential targets of physiologically important miRNAs in a non-model organism.

## Author summary

Metabolic homeostasis is critical for the fundamental physiological processes of organisms, and its dysregulation leads to severe metabolic and physiological diseases, such as obesity and insulin resistance. miRNA is a type of endogenous non-coding RNA that participates in multiple biological processes directly or indirectly. In the present study, we found the involvement of the miR-275/305 cluster in energy metabolism in female *B*. *dorsalis* and revealed the related post-transcriptional regulatory mechanisms. The miR-275/305 cluster expression positively responded to dietary yeast stimulation, and its knockdown had a profound impact on the triglyceride, glycogen, and glucose metabolisms at the adult stage of *B*. *dorsalis*. Furthermore, we uncovered that the miR-275/305 cluster mainly functions through negatively regulating the transcription of the *SLC2A1/GLIS2*, eventually ensuring normal metabolic physiology. Further, this miRNA-target axis was under the control of the conserved insulin signaling pathway. Therefore, our findings provide novel insight into the molecular basis of the metabolic adaptation to nutrient availability in *B*. *dorsalis*.

## Introduction

Throughout the life-cycles of insects, it is necessary to continuously obtain nutrients for normal growth and development of larvae and reproduction of adults. Adequate nutrient intake can guarantee the survival of individuals and the continuation of populations. Protein and carbohydrate are two major components of the insect diet, and an imbalanced carbohydrate-to-protein ratio has a profound impact on age-dependent survival and reproductive fitness [1, 2]. In many dipterous insects, newly eclosed adults must consume a certain amount of protein to reach sexual maturity and reproduce offspring. As a compound nutrient source, yeast extract mainly contains nitrogen sources, vitamins, and trace minerals, which can provide favourable and balanced nutrients for insect development and production, and is widely used for the culture of insects of the order Diptera. Studies across different species have indicated that protein rather than carbohydrate is the determining factor in controlling larval development, metamorphosis, reproduction, longevity and copulatory success [3–7].

In addition, cellular metabolism is sensitive to nutrition fluctuations and requires metabolic adaption to the dynamic changes in available nutrients [8]. In *Drosophila melanogaster*, increased amounts of yeast extract can suppress fly adiposity in the presence of low dietary sugar, indicating that dietary yeast has a strong suppression effect on triglyceride (TAG) storage [4]. High yeast extract levels lead to severe disruption of metabolic homeostasis and the induction of an insulin-resistant phenotype [9]. Somatic storage of metabolic substances is also largely dependent upon the level of dietary yeast available to flies.

Precise control of metabolic homeostasis is a fundamental physiological process, which is critical for cellular differentiation and tissue integrity in multicellular organisms [8]. Although considerable studies have reported the essential role of dietary yeast in physiological metabolic process, the concerning metabolic homeostasis regulation mechanisms are highly complex and remain incompletely understood. Increasing evidence indicates that dietary components could reshape the miRNA expression profile of organisms, leaving the possibility that dietary yeast may modulate metabolism via microRNA (miRNA) [10–12].

miRNA is a type of non-coding nucleotide sequence with a length of about 21 nt, and it can inhibit or promote gene expression at the post-transcriptional level by binding to the 3’UTR of the gene [13, 14]. Dysfunction of the miRNA-target regulatory network has been related to systemic metabolic disturbance and hormonal imbalance [15]. For example, *Drosophila* miR-14 acts as a tumor suppressor, inhibiting TAG accumulation and alleviating cell death rates [16]. Lacking miR-278 leads to elevated circulating sugar levels and exhibits an insulin resistance phenotype [17]. miR-210 functions to regulate lipid metabolism and protect the retina from neurodegeneration [18]. In *Aedes aegypti*, the genetic depletion of miR-277 results in lipid accumulation defects and ovarian development stasis [19]. miR-276 contributes to the shifts from catabolic metabolism to anabolic metabolism in *Anopheles coluzzii* [20]. Collectively, these findings suggest that miRNAs acting as transcription modulators are widely involved in metabolic processes.

*Bactrocera dorsalis* is a notorious horticultural pest that can damage more than 350 fruits and vegetables [21, 22]. A detailed understanding of the fundamental biological processes in *B*. *dorsalis*, such as metabolism, can provide a solid theoretical basis for developing effective pest control strategies. Although genome-wide transcriptomic data has revealed that different nutritional diets can regulate multiple mRNAs participating in metabolic pathways [23–25], little is known about the underlying molecular mechanisms.

In the present study, we found that both yeast consumption and interference with miRNA biosynthesis pathway genes can significantly affect the metabolic status of adult flies. By small RNA sequencing, we identified a conserved miR-275/305 cluster, which displayed strong responsiveness to dietary yeast stimulation. Knockout of miRNA-275/305 induced metabolic abnormalities and impaired flight ability. Combined *in vitro* and *in vivo* experiments demonstrated that miR-275 and miR-305 can suppress *SLC2A1* and *GLIS2* expressions to maintain metabolism homeostasis under yeast-rich diet conditions. Further studies revealed that this miRNA-target axis is regulated by the insulin signaling pathway.

## Results

### Consumption of dietary yeast affects the metabolism status of adult *B. dorsalis*

**Fig 1:**
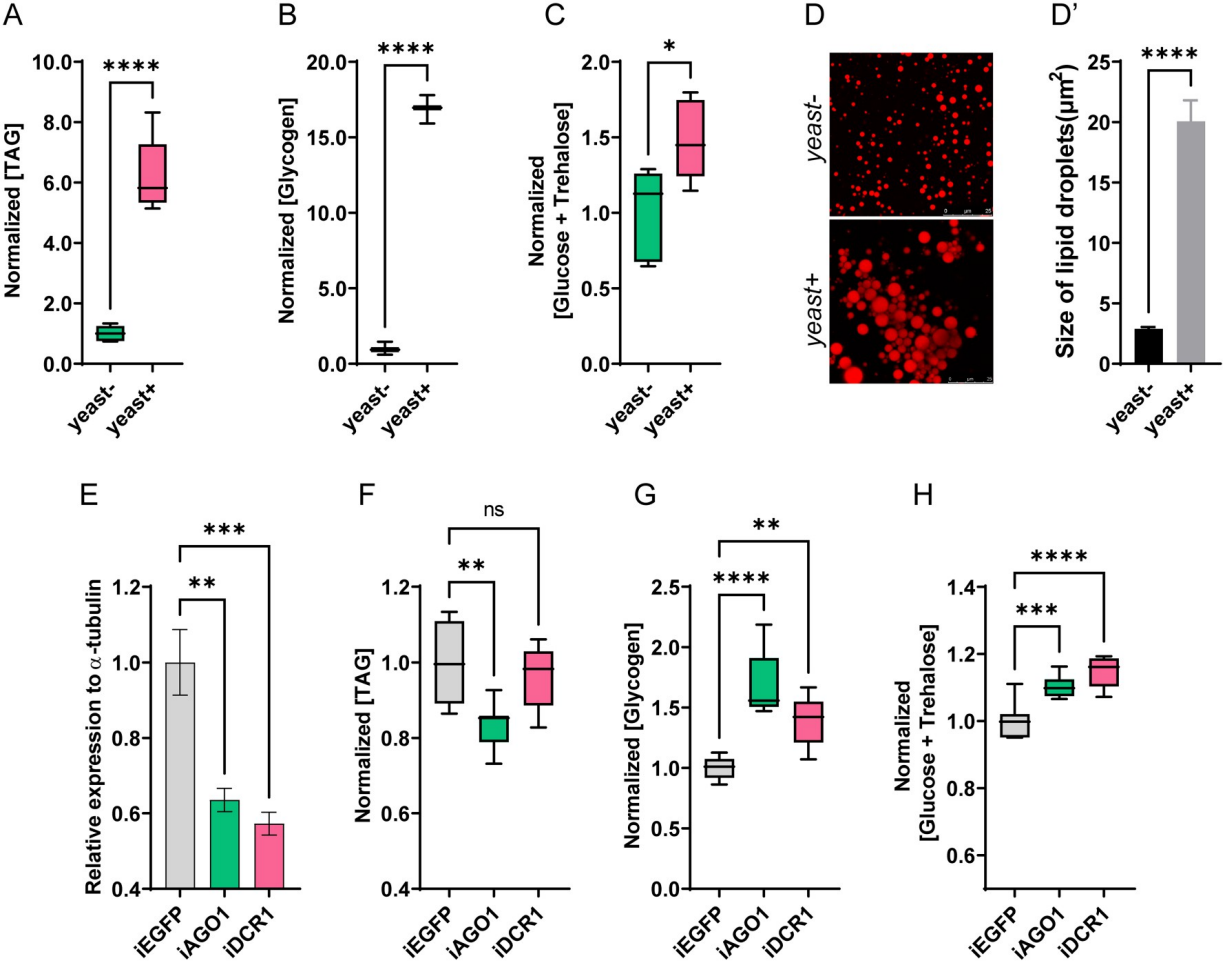
Dietary yeast may influence metabolic status via miRNA. The female of *B*. *dorsalis* were fed with or without dietary yeast for 4 days. Whole bodies of flies were homogenized in PBST solution, and then the contents of TAG (A), glycogen (B) or total sugar content (glucose plus trehalose) (C) were detected. The results were normalized to the total protein content. Boxplots show the data of seven independent biological replicates (n = 5 flies for each replicate). Asterisks indicate significant differences by Student’s *t*-test (****, *P* < 0.0001; *, *P* < 0.05). (D-D’) Nile-red staining of the lipid droplets in the fat body of females. Pictures were obtained with a Leica SP8 confocal microscope (Scale bar: 20 μm), and the lipid droplet size was quantified using ImageJ. “yeast-” and “yeast+” indicate the absence and presence of yeast in the diet, respectively. (E) RNAi efficiency of miRNA biosynthesis pathway-related genes *AGO1* and *DCR1* at 48 h post dsRNA microinjection. Data are expressed as means ± SEM of four independent replicates (***, *P* < 0.001; **, *P* < 0.01). (F-H) Effects of *AGO1* and *DCR1* RNAi on the metabolic content. (G) TAG content. (H) glycogen content. (I) total sugar content (glucose plus trehalose).

Available nutrition is an important environmental constraint that influences multiple developmental processes in insects, especially nutrition-sensitive metabolism [8]. The imbalance between energy intake and energy expenditure is related to metabolic disturbances. To evaluate the effect of dietary yeast on adult metabolism, we first quantified the metabolites of wild-type flies in response to different dietary treatments for four days. These results showed that consuming a yeast-rich diet can significantly increase total TAG and glycogen content in the whole bodies, 6.2 and 16.9 times more than the control group, respectively, while the total sugar content (glucose plus trehalose) had a slight increase (Fig 1A–1C). Since TAG is mainly stored in the fat body as lipid droplets, we next evaluated the changes in the lipid droplet size in these flies using Nile Red staining. As shown in Fig 1D, the average size of lipid droplets in the yeast-rich group was significantly larger than that of the control group (Fig 1D and 1D’). Overall, consumption of dietary yeast has a substantial effect on the metabolic status of adult *B*. *dorsalis*.

### Dietary yeast may modulate metabolism via miRNAs

Increasing evidence has revealed that diet can also regulate miRNA expression in addition to affecting gene transcription. miRNAs, as important post-transcriptional regulators of genes, are involved in a variety of physiological processes, including metabolism [15]. Therefore, we asked whether the consumption of dietary yeast affects adult metabolic physiology via miRNA. Since *AGO1* and *DCR1* are the main effectors of the miRNA biosynthesis pathway [26, 27], we first investigated the effect of the silencing both genes on adult metabolic physiology. The knockdown efficiency by RNAi was shown in Fig 1E (*iAGO1* vs. *iEGFP*, *P* = 0.0033; *iDCR1* vs. *iEGFP*, *P* = 0.0004). The measurement of metabolites showed that the knockdown of *AGO1* resulted in a significant decrease in TAG content compared with *dsEGFP* control, whereas the knockdown of *DCR1* led to no substantial change (*iAGO1* vs. *iEGFP*, *P* = 0.004; *iDCR1* vs. *iEGFP*, *P* = 0.504). In contrast, the contents of glycogen and total sugar were significantly higher in *AGO1* and *DCR1* knockdown groups than in the control group (glycogen: *iAGO1* vs. *iEGFP*, *P* < 0.0001; *iDCR1* vs. *iEGFP*, *P* = 0.0016; glucose plus trehalose: *iAGO1* vs. *iEGFP*, *P* = 0.0007; *iDCR1* vs. *iEGFP*, *P* < 0.0001) (Fig 1F–1H). Therefore, it could be concluded that miRNA might be involved in the process of dietary yeast-induced metabolic changes in *B*. *dorsalis*.

### Identification of miRNAs potentially involved in energy metabolism

**Fig 2:**
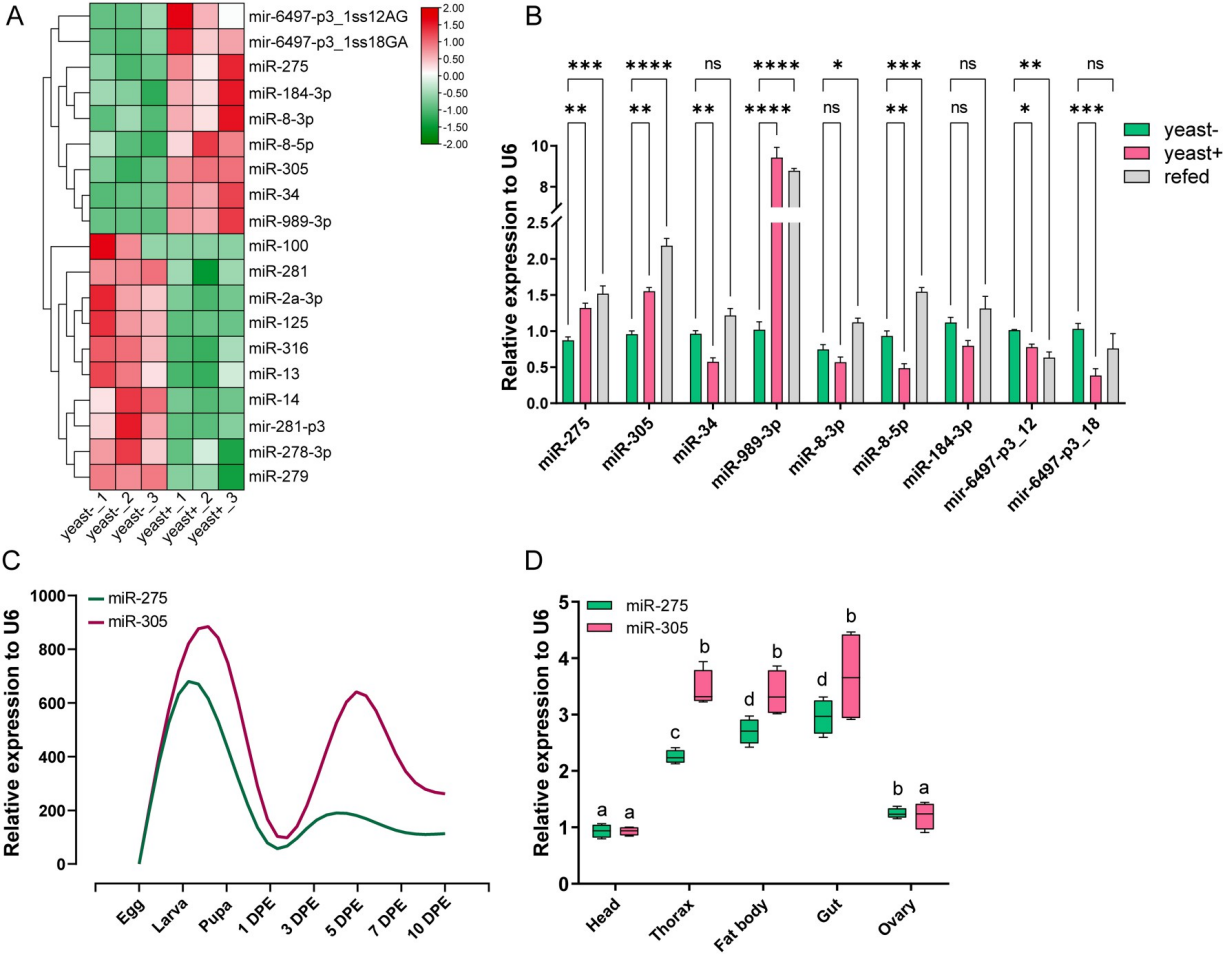
Identification of miRNAs potentially involved in energy metabolism in *B*. *dorsalis*. (A) Hierarchical clustering analysis of the differentially expressed miRNAs in response to dietary yeast stimulation. Heatmap showed two separated clades with up-regulated miRNAs in one clade and down-regulated miRNAs in the other clade. The color scale indicates the Log2-transformed expression values in the heatmap. (B) Expression levels of up-regulated miRNAs in the abdomens of *B*. *dorsalis* treated with three different dietary regimens, namely, yeast-free diet (yeast-), yeast-rich diet (yeast+), and 4-day yeast-free diet followed by 2-day yeast-rich diet (refed). Data are expressed as means ±SEM. ****, *P*< 0.0001; ***, *P* < 0.001; **, *P* < 0.01. (C-D) Relative expression levels of miR-275 and miR-305 during the larvae-pupa-adult developmental stages (C) and different tissues isolated at 5 day post eclosion (DPE) of the adult female (D). The smooth curve was fitted using the LOWESS spline method with GraphPad Prism to visualize the dynamic expression of miRNA. Different lowercase letters above bars denote significant differences (*P* < 0.05) according to Tukey’s test (one-way ANOVA).

To identify potential miRNAs involved in metabolic processes, six cDNA libraries from two different dietary treatments (yeast+ vs. yeast-) were constructed and subjected to small RNA sequencing analysis. A total of 19 differentially expressed miRNAs were identified with an expression cutoff value greater than average and a false discovery rate of < 0.05 (Fig 2A). We pay special attention to these up-regulated miRNAs in the yeast-rich group since they positively respond to dietary yeast stimulation. Next, we measured the expression of these significantly up-regulated miRNAs using the re-feeding assay to validate the stimulating effect of dietary yeast. The qRT-PCR results showed that among the 9 up-regulated miRNAs, only miR-275, miR-305 and miR-989-3p were significantly lower expressed in the yeast-free group compared to the yeast-rich group, and vigorously elevated to the yeast-rich level following yeast re-feeding (Fig 2B). miR-989-3p has been reported to be specifically expressed in the ovary [28–30], and is involved in border cell migration in *Drosophila* [31]. miR-275 and miR-305 were identified as adjacent miRNA within a cluster and exhibited a synergistic response to protein-rich diet stimulation [32] [33]. The sensitive response to dietary yeast implied that they might be involved in the process of the dynamic changes of metabolism, and thus we focused on these two unexplored miRNAs in the following study.

Before implementing functional analysis, we first examined the spatial and temporal expression patterns of miR-275 and miR-305 using qRT-PCR. A time-course expression profile indicated that both were significantly enriched in larval and pupal stages, and miR-305 exhibited another peak at 5 days post eclosion (DPE). The tissue distribution results showed that miR-275 and miR-305 were highly expressed in fat-rich tissues such as the gut and fat body (Fig 2C and 2D), which are critical tissues for metabolic processes [34, 35]. Therefore, the above results suggest that the miR-275/305 cluster might play a role in regulating nutrition utilization and energy metabolism.

### miR-275 and miR-305 mutations cause severe energy metabolism defects

**Fig 3:**
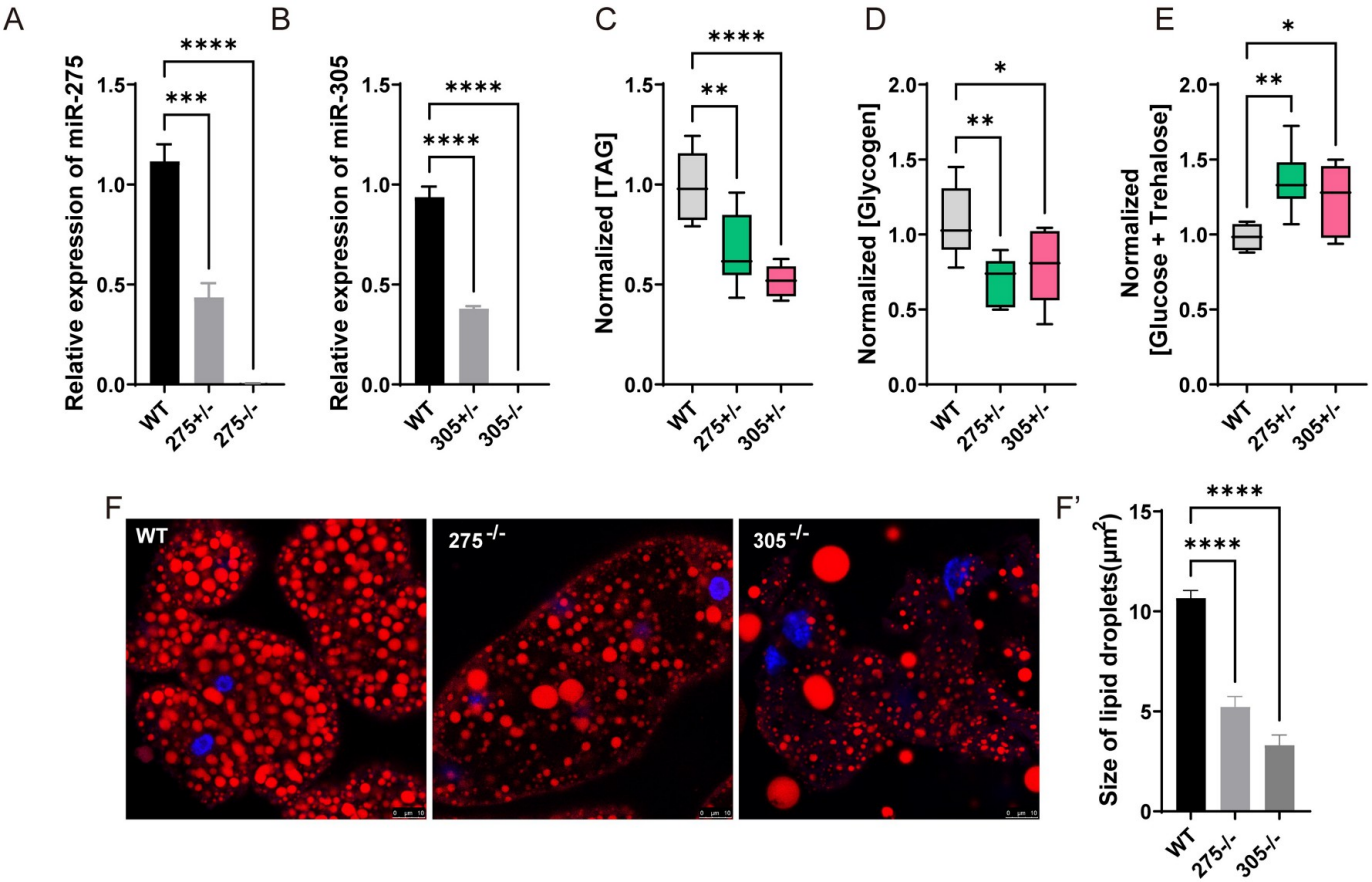
Targeted mutation of miR-275 and miR-305 impaired physiological metabolism. (A-B) Relative expression levels of mature miR-275 (A) and miR-305 (B) in homozygote (-/-), heterozygote (+/-), and wild-type flies. (C-E) Effects of miRNA mutation on energy substrate metabolism in heterozygote. (C) TAG content. (D) glycogen content. (E) total sugar content (glucose plus trehalose). Data were normalized to total protein content. Data represent seven to ten biological replicates with three technical replicates. *, *P* < 0.05; **, *P* < 0.01; ***, *P* < 0.001, and ****, *P* < 0.0001 (Student’s t-test). (F-F’) Nile Red staining of lipid droplets. The fat body was obtained from *miR-275*^*-/-*^, *miR-305*^*-/-*^, and wild-type females at 5 DPE. Red, Nile Red staining for neutral lipids; and blue, DAPI staining for nuclei. Lipid droplets were visualized and imaged with a Leica SP8 confocal microscope (Scale bar: 10 μm.), and the size was quantified using ImageJ.

To further explore the physiological functions of the miR-275/305 cluster, we generated miR-275 and miR-305 mutants using the CRISPR/Cas9 system, the different types of insertion or deletion mutations were shown in S1 Fig. qRT-PCR indicated that the mature miRNA level in miR-275 and miR-305 heterozygous mutants (hereafter termed as *miR-275*^+/-^ and *miR-305*^*+/-*^) was 0.39 and 0.40 folds of that in wild-type flies, respectively (Fig 3A and 3B). Homozygous mutants were semi-lethal, and only 4.46%-14.75% survived to adulthood (S1 Table). Further, there was no fertile offspring generated from homozygous crossing. Therefore, the following experiments were mainly performed using heterozygous mutants. Metabolism assay results showed that the TAG content in *miR-275*^+/-^ and *miR-305*^*+/-*^ was decreased to 68.4% and 51.9% of that in wild-type flies, respectively (Fig 3C). The glycogen content in *miR-275*^*+/-*^ and *miR-305*^*+/-*^ was about 64.6% and 70.6% of that in wild-type flies, respectively (Fig 3D). In contrast, the depletion of miR-275 and miR-305 significantly elevated total sugar content by 39.0% and 28.7%, respectively, compared with that in wild-type flies (Fig 3E). Consistent with the *in vitro* assay results, Nile Red staining results showed that the lipid droplet size of mutant flies was smaller than that of the wild-type flies at 5 DPE (Fig 3F and F’). Together, these results suggest that miR-275 and miR-305 are essential for maintaining metabolic balance.

### Mutations of miR-275 and miR-305 influence fitness-related traits of *B. dorsalis*

**Fig 4:**
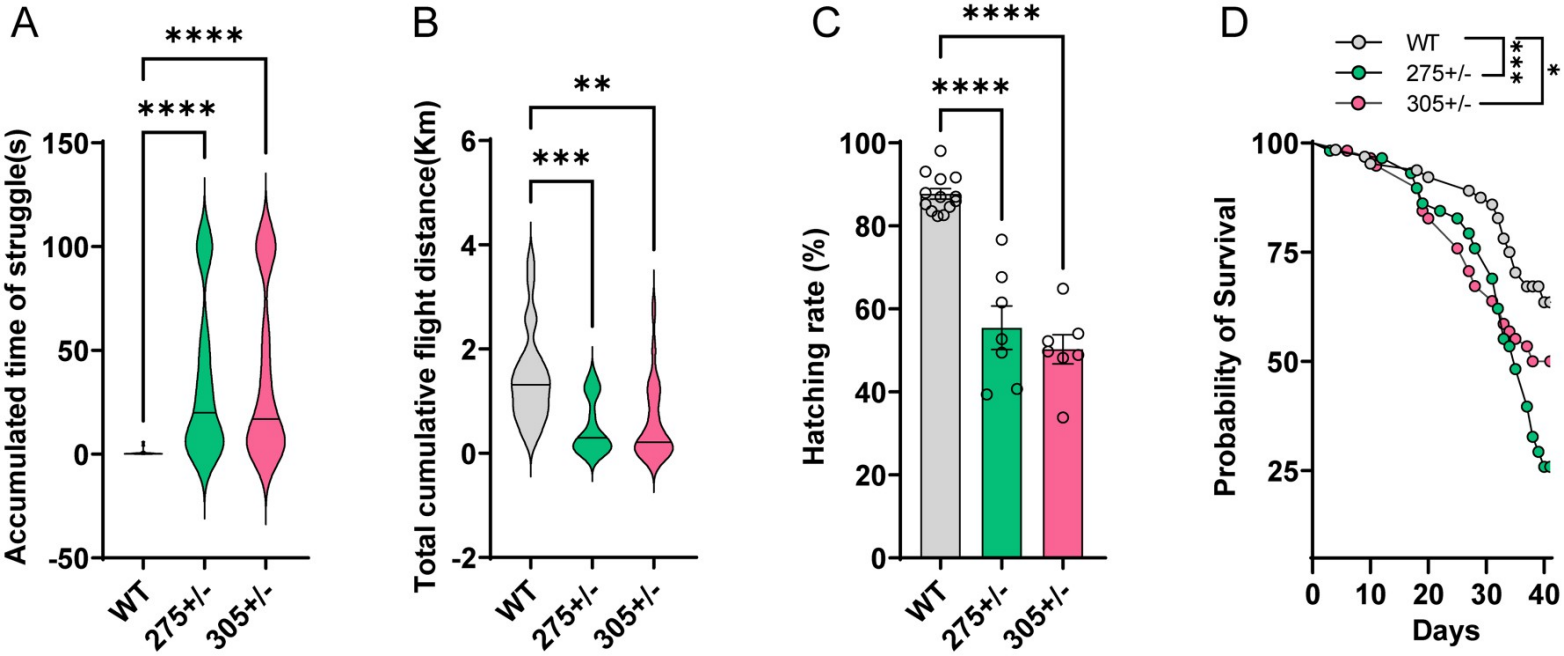
miR-275 and miR-305 maternal loss-of-function mutants exhibited impaired flight capability, decreased hatching rate and survival rate. (A) Negative flip duration of heterozygous mutants and wild-type flies. (B) Effect of miRNA mutation on the cumulative flight distance. The flight mill system was used to compute the flight performance of heterozygote mutant and wild-type females at 10 DPE. (C) Embryo hatchability of the heterozygous mutants and wild-type flies. Flies with depleted miR-275 and miR-305 showed only 55.48% and 50.28% mean hatching rates, whereas wild-type flies showed an 87.74% mean hatching rate. Each treatment contained 200 eggs and 8 biological replicates. (D) Survival curves of heterozygous mutant and wild-type adult female flies. The survival rate was monitored for 40 days after genotype identification. Survival curves indicated that the survival rate of *miR-275*^*+/-*^ and *miR-305*^*+/-*^ was significantly lower than wild-type flies (*P* = 0.0002 and *P* = 0.037, respectively, n = 60, Gehan-Breslow-Wilcoxon test). The data are expressed as mean ± SEM. ***, *P* < 0.001.

We noticed that mutants exhibited motor impairment. Some severely impaired mutants were prone to turning upside down during climbing and struggled to turn over for a long period, which displayed a similar symptom of muscle weakness (S1–S3 Movies). To quantify the negative flip ability, the flies’ behaviors were tracked using electronic video recording devices. When their tergum faced the ground, wild-type flies responded rapidly and turned over within less than 2 seconds, whereas mutant flies failed to do so, and it took more than 3 minutes for some severely impaired mutant flies to turn over (Fig 4A, *P* < 0.0001 for the *miR-275*^+/-^, and *P* < 0.0001 for the *miR-305*^+/-^). The insect flight capability assay showed that the mutant flies exhibited a significantly lower cumulative flight distance than wild-type flies (Fig 4B, *P* = 0.0007 for the *miR-275*^+/-^, and *P* = 0.0013 for the *miR-305*^+/-^). In addition, mutants exhibited lower survival rate, hatching rate, and eggshell biogenesis defects than wild-type flies (Fig 4C and 4D, and S2A and S2B Fig), indicating that this miR-275/305 cluster might be also involved in ageing and eggshell secretion. Collectively, these results suggest that loss of miR-275/305 profoundly affects systemic metabolic homeostasis, thus compromising flight performance.

### Candidate target genes are identified

**Fig 5:**
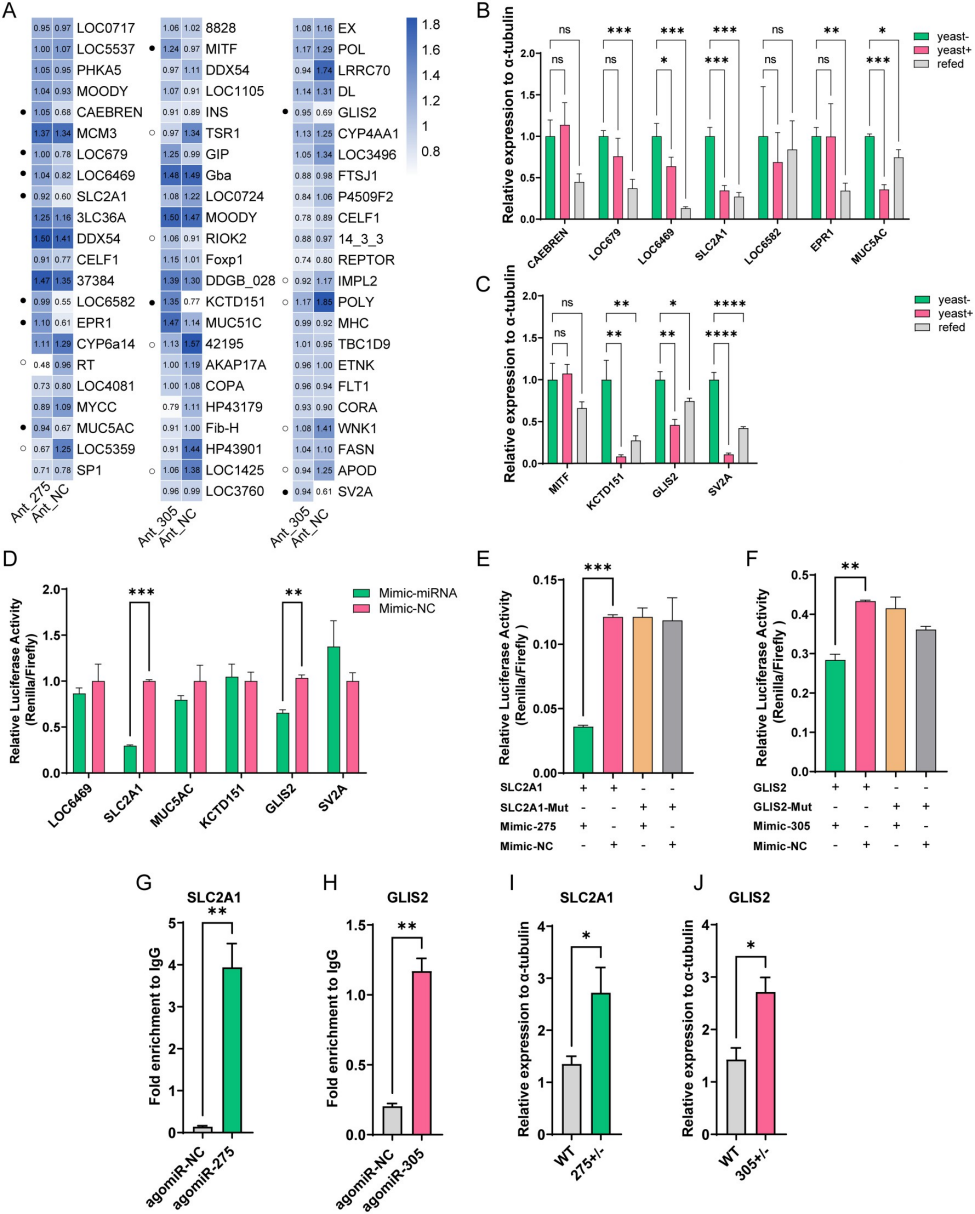
SLC2A1 and GLIS2 are the direct targets of miR-275 and miR-305, respectively. (A) Heatmap of relative expression levels of candidate targets in antagomiR groups (Ant-275 or Ant-305) and the negative control (Ant-NC). The number in each cell represents the mean value of the relative expression. Solid and hollow circles indicate significantly up-regulated and down-regulated target genes upon antagomiR treatment, respectively (*P* < 0.05). (B-C) Validation of candidate target genes by yeast-refeeding strategy. (B) miR-275. (C) miR-305. “yeast-” and “yeast+” indicate the absence and presence of yeast in the diet for 6 days, respectively, while “refed” indicates a 4-day yeast-free diet followed by a 2-day yeast-rich diet (refed). ANOVA: *, *P* < 0.05; **, *P* < 0.01; ***, *P* < 0.001. (D) Luciferase reporter assays of candidate targets (*LOC6469*, *SLC2A1*, *MUC5AC*, *KCTD151*, *GLIS2*, and *SV2A*) *in vitro*. (E-F) Abolishment of repression effect by binding site mutation. (E) miR-275-SLC2A1 binding site mutation. (F) miR-305-GLIS2 binding site mutation. (G-H) Immunoprecipitation (RIP) of miRNA and their target genes *in vivo*. (G) RIP of miR-275 and *SLC2A1*. (H) RIP of miR-305 and *GLIS2*. Fold enrichment was quantitated relative to IgG control. (I-J) Relative expression of *SLC2A1* and *GLIS2* in the heterozygous mutant and wild-type flies. Data are expressed as means ± SEM. *, *P* < 0.05; **, *P* < 0.01; ***, *P* < 0.001; and ****, *P* < 0.0001 (Student’s t-test).

To better understand how miR-275 and miR-305 modulate metabolic physiology, genome-wide transcriptional analysis was conducted to identify the potential target genes [36–38]. We obtained 22 and 46 candidate targets for miR-275 and miR-305 by target predictions and the omic-data-based association analysis (S2 Table). Subsequently, we employed the antagomiR to knock down miR-275 and miR-305 and measured the relative expression changes of these potential target genes by qRT-PCR. The antagomiR knockdown efficiency and phenotypic manifestation were confirmed and shown in S3A–S3E Fig. A total of 9 (Ant-275 vs. Ant-NC) and 12 (Ant-305 vs. Ant-NC) differentially expressed candidate target genes were obtained (*P* < 0.05), respectively (Fig 5A). Since miRNA mainly negatively regulated the transcription of genes, only significantly up-regulated target genes were further analyzed.

Considering that the miR-275/305 cluster responded to dietary yeast stimulation, we speculated that target genes might also be sensitive to dietary yeast. We then employed the yeast re-feeding method to verify changes of target genes by qRT-PCR. Among the examined candidates, the transcript levels of *LOC6469*, *MUC5AC*, and *SLC2A1* of miR-275 were high in the yeast-free diet, and then returned to those of the yeast-rich diet after re-feeding. The potential targets of miR-305, including *KCTD151*, *GLIS2*, and *SV2A*, also showed a similar expression trend in the re-feeding test (Fig 5B and 5C). Meanwhile, the sequence alignment revealed that the 3’UTR of these candidate target genes contained putative binding sites with typical perfect match of the 2–7 nt miRNAs’ seed sequences (S4 Fig). The above results suggest that these targets might be candidate genes for miRNA to function.

### SLC2A1 and GLIS2 are the direct target of miR-275 and miR-305, respectively

To validate whether miR-275 and miR-305 could degrade these target genes or inhibit their translation, dual-luciferase reporter assays were conducted in HEK-293T cells to illustrate their potential interactions *in vitro*. After co-transfection of gene fusion recombinant plasmid and the miRNA mimic, only *SLC2A1* and *GLIS2* reduced the relative luciferase activity by 70.4% and 34.5%, compared with control (mimic-NC), respectively, whereas the other target genes failed to inhibit the luciferase activity (Fig 5D). Conversely, mutating binding sites complementary to the seed sequences abolished the inhibition of the luciferase activity in mutant constructs, indicating that the miR-275 and miR-305 might bind directly to the *SLC2A1* and *GLIS2* to inhibit their expression, respectively (Fig 5E and 5F).

To further confirm this binding and its specificity, we performed RNA immunoprecipitation (RIP) experiments with antibodies against *B*. *dorsalis* Argonaute 1 (AGO1), which is the central component of the RNA-induced silencing complex (RISC). The results showed that *SLC2A1* or *GLIS2* were significantly enriched (28.2 folds and 5.7 folds, respectively) in the AGO1-immunoprecipitated RNAs from the abdomens treated with agomiR-275 or agomiR-305 compared with those treated with agomiR-NC (Fig 5G and 5H). Subsequently, enhanced transcription levels of *SLC2A1* and *GLIS2* were also detected in the *miR-275*^*+/-*^ and *miR-305*^*+/-*^ mutants (Fig 5I and 5J). Collectively, these results confirm that miR-275 and miR-305 are directly bound to the 3’UTR of *SLC2A1* and *GLIS2* mRNA to repress their expression, respectively.

### Co-localization of miRNAs and their targets

**Fig 6:**
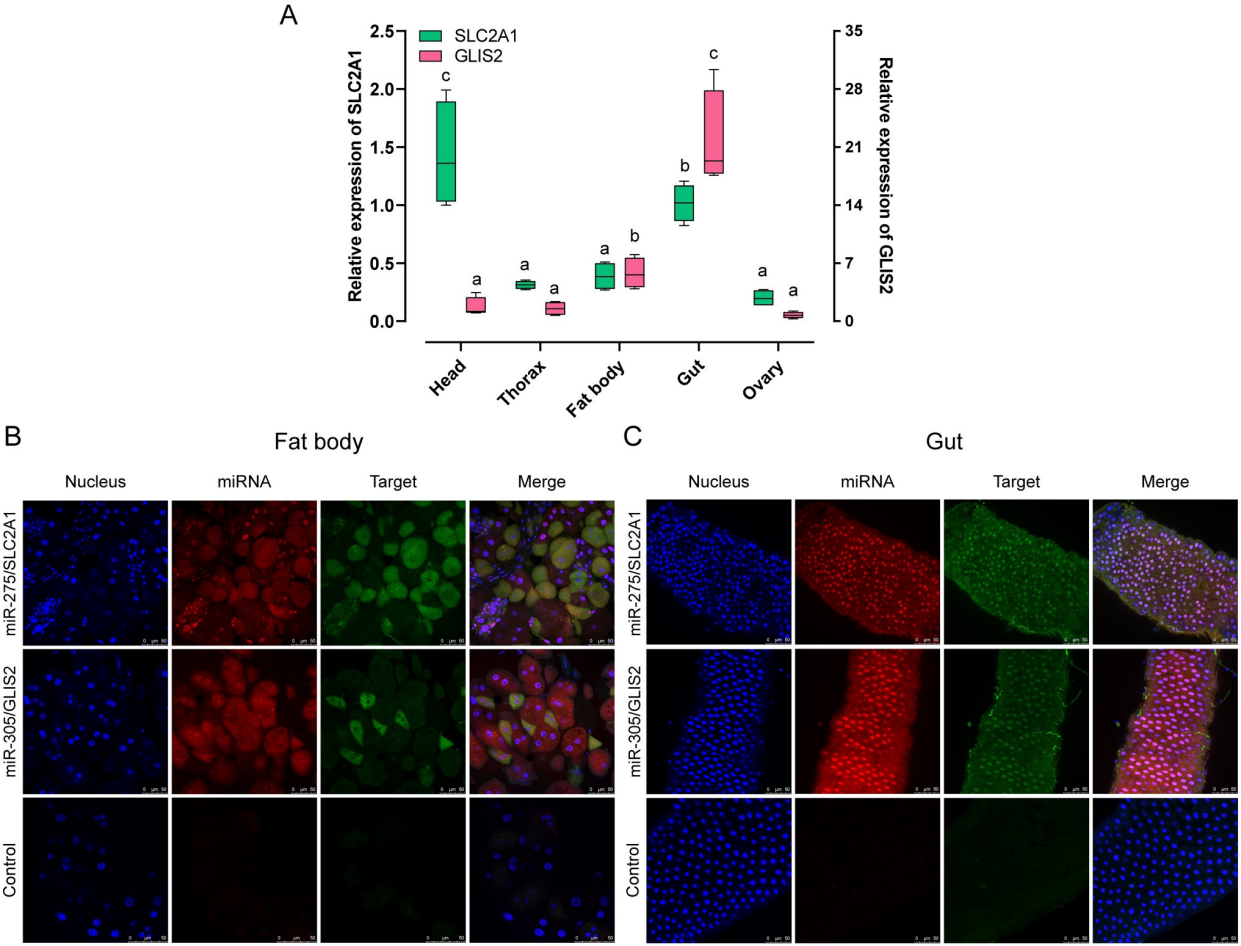
Tissue-specific expression profile of target genes and RNA in situ hybridization. (A-B) Tissue-specific expression patterns of *SLC2A1* (A) and *GLIS2* (B) with 5-day-old females. Data are expressed as mean ± SEM from five biological replicates. The lowercase letters above bars indicate significant differences by one-way ANOVA followed by Tukey’s multiple comparisons test. (C-D) RNA-FISH detection of miR-275/miR-305 (Red) and *SLC2A1/GLIS2* (Green) in the fat body (C) and gut (D) of wild-type flies. The images were visualized using Leica TCS SP8 confocal laser scanning microscope (Zeiss, Germany) at a magnification of 40 x. Scale bar: 50 μm.

miRNA regulates target gene expression in a spatio-temporal and tissue-specific manner [13, 39]. The relatively high expression of miR-275 and miR-305 in the fat body and gut inspired us to verify whether their target genes were also highly expressed in these tissues. For this purpose, the expression patterns of target genes in different tissues of 5-day-old adults were examined. qRT-PCR results indicated that *SLC2A1* and *GLIS2* were widely expressed in various tissues, especially in the gut, head, and fat body, which was similar to the expression of miRNAs (Fig 6A).

To further determine whether miRNAs were co-localized with their targets in these tissues, we performed fluorescence in situ hybridization (FISH) assays using specific RNA probes. The results showed that miR-275 and *SLC2A1* specific fluorescent signals were co-localized in the fat body cells and intestinal epithelial cells, as were miR-305 and *GLIS2* (Fig 6B and 6C). Thus, miR-275 and *SLC2A1* as well as miR-305 and *GLIS2* co-localizations in the gut and fat body confirmed their direct interactions in a spatial dependent manner.

### RNA interference of SLC2A1 and GLIS2 partially rescues metabolic defects

**Fig 7:**
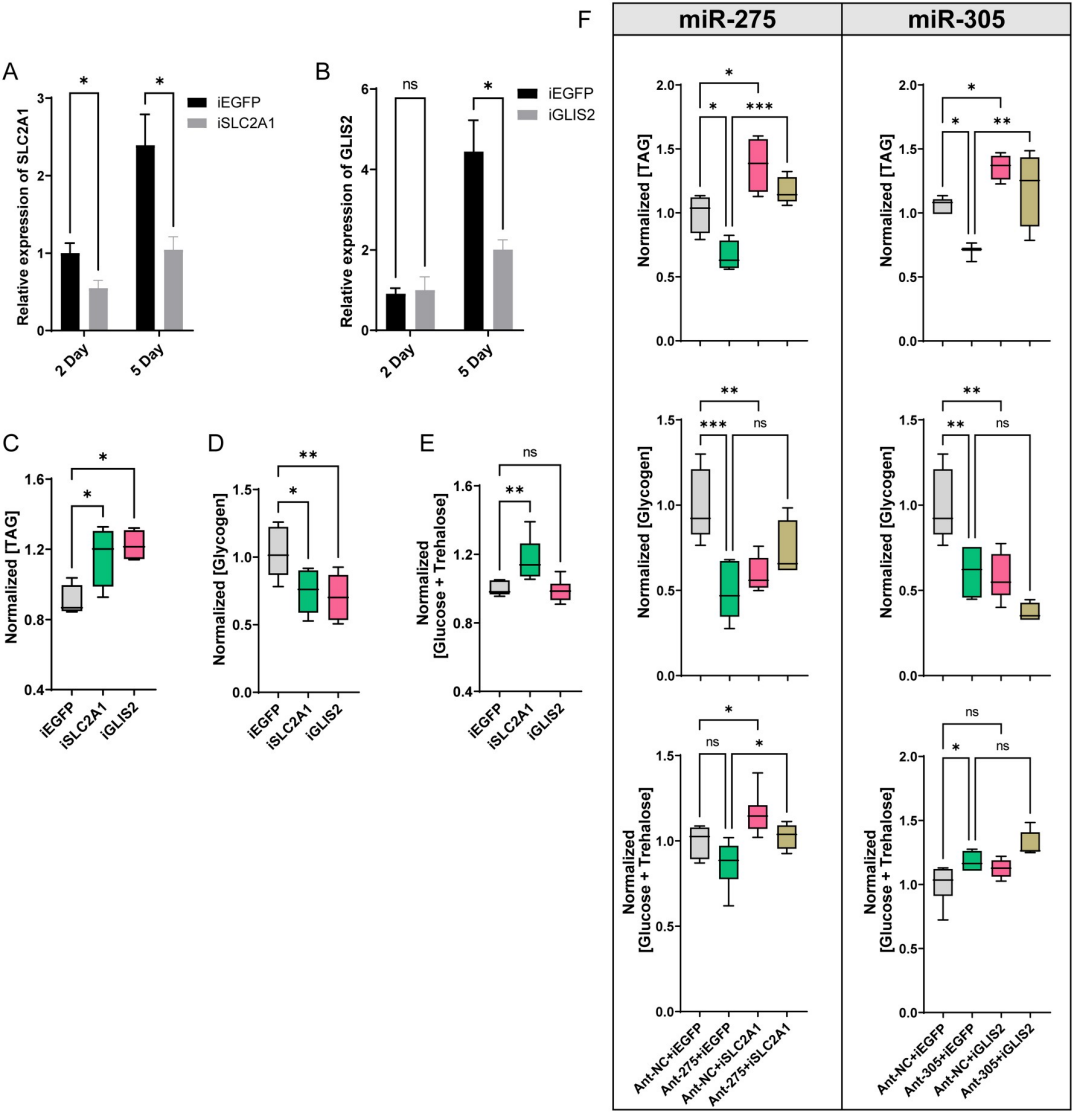
Knockdown of SLC2A1/GLIS2 partially rescues metabolic phenotype defects in antagomiR-treated flies. (A-B) RNAi knockdown efficiency of *SLC2A1* and *GLIS2* at 2 days and 5 days post dsRNA injection by qRT-PCR. (C-E) Effects of dsRNA treatment on the contents of TAG (C), glycogen (D), and total sugar (glucose plus trehalose) (at 5 days post-injection) (G). (F) Metabolic phenotypes of antagomiR-treated flies in rescue experiments. The experiments were performed by co-injecting dsRNA and antagomiR with co-injection of *dsEGFP* and the antagomiR-control (Ant-NC) as a negative control. Left panel represents the rescue for miR-275 deletion, and right panel for miR-305 deletion. Data represent 10 biological replicates for each sample. One-way ANOVA was performed to determine statistically significant differences. *, *P* < 0.05; **, *P* < 0.01; ***, *P* < 0.001, NS, not significant.

Functional analysis indicated that *SLC2A1*, previously known as solute carrier family 2 member 1, is responsible for constitutive or basal glucose uptake [40]. The increase of *SLC2A1* tends to be accompanied by an increase in cellular glucose uptake [41, 42]. Knockdown of *SLC2A1* can significantly restrain cell glycolysis and proliferation [43, 44]. *GLIS2* is a Krüppel-like zinc finger transcription factor, belonging to the Gli-similar (Glis) subfamily, and it functions as a transcription factor to suppress multiple lipase genes involved in fat catabolism [24, 45]. In order to confirm whether *SLC2A1* and *GLIS2* were involved in the metabolic process in *B*. *dorsalis*, we performed RNAi-mediated knockdown of target genes separately. The qRT-PCR results showed that the expression levels of *SLC2A1* and *GLIS2* were significantly decreased to 43.7% and 45.2% of the *dsEGFP* control at 5 days post dsRNA injection (Fig 7A and 7B). Subsequently, metabolism assay revealed that the content of TAG in the *dsSLC2A1* and *dsGLIS2* treatment groups was significantly higher than that in the *dsEGFP* treatment group (*iSLC2A1* vs. *iEGFP*, *P* = 0.0292; *iGLIS2* vs. *iEGFP*, *P* = 0.0101), and *SLC2A1* and *GLIS2* knockdown resulted in a significant decrease in the glycogen content (*iSLC2A1* vs. *iEGFP*, *P* = 0.0164; *iGLIS2* vs. *iEGFP*, *P* = 0.0046). In addition, an increase in approximately 55% of sugar content was observed only in the *dsSLC2A1* group, compared to *dsEGFP (iSLC2A1* vs. *iEGFP*, *P = 0*.*0082)* (Fig 7C–7E).

Although the depletion of miRNA or target genes both resulted in energy metabolic disorders, whether these metabolic phenotypes under antagomiR treatment were caused by increased expression of *SLC2A1* and *GLIS2* remains unclear. Therefore, we conducted phenotypic rescue experiments by silencing *SLC2A1* and *GLIS2* after sample antagomiR treatment. Indeed, lipid metabolic defects resulting from the miR-275 and miR-305 inhibition were successfully rescued after silencing their main target genes *SLC2A1* and *GLIS2* by RNAi, respectively (Fig 7F). The mixed injection with antagomiR and dsRNA did not significantly affect the contents of glycogen and the total sugar (Fig 7F). In conclusion, these results suggest that RNAi interference with the major target genes *SLC2A1* and *GLIS2* could partially rescue these metabolic phenotypes.

### miRNA-targets are regulated by the insulin signaling pathway in a nutrient-dependent manner

**Fig 8:**
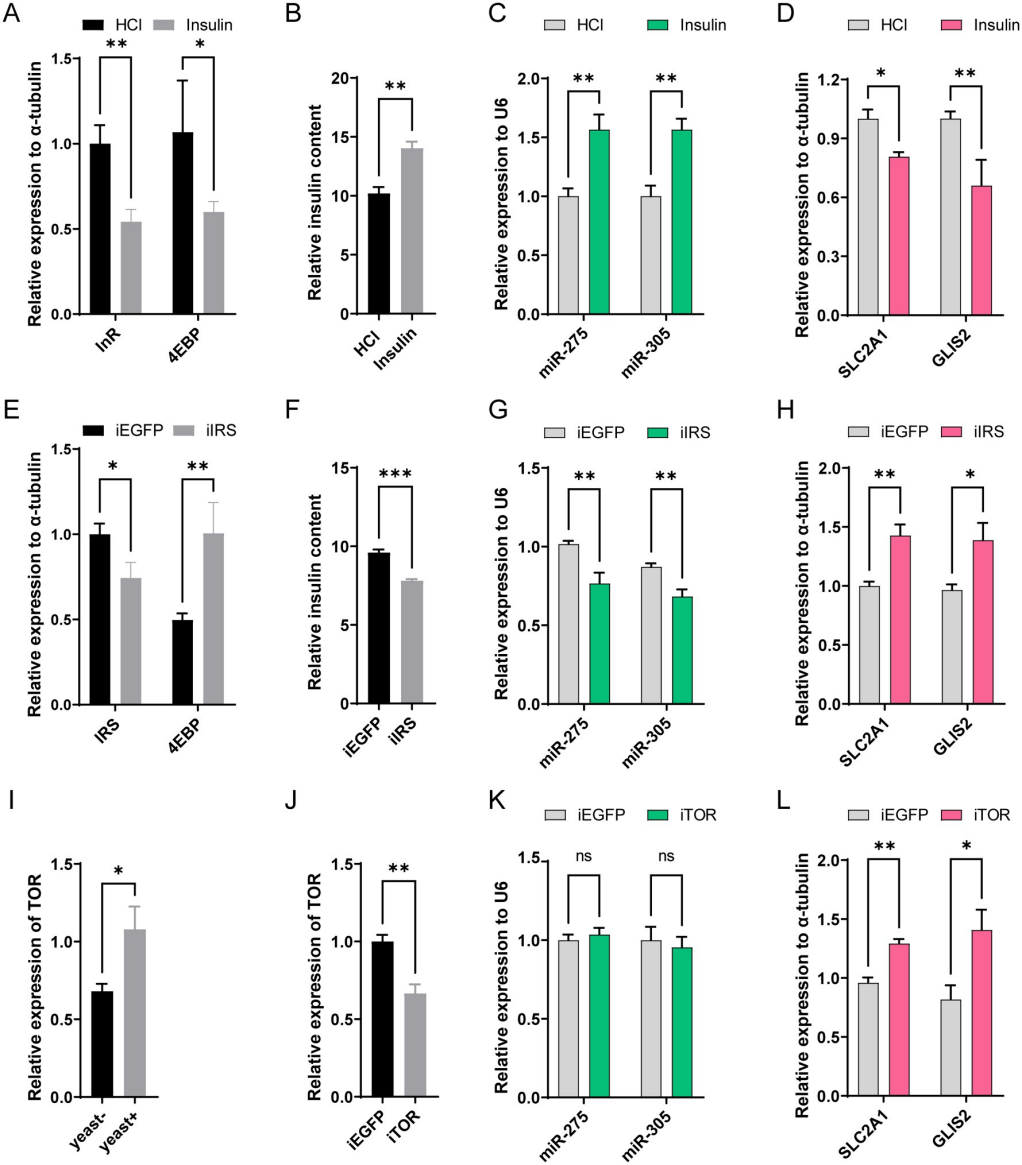
The insulin pathway is involved in the regulation of the miRNA-target axis. (A-D) Effects of enhanced insulin activity on the miRNA-target axis under yeast-rich conditions (yeast+). (A) The relative transcription levels of *InR* and *4EBP* at 24 h post insulin injection. (B) ELISA was employed to quantify the relative insulin content, including the sum of exogenous insulin and insect-produced insulin. (C-D) The relative expression levels of miRNAs (C) and target genes (D) following insulin microinjection. (E-H) Effects of inhibiting the insulin signaling pathway gene *IRS* on the miRNA-target axis. (E) The relative expression levels of *IRS* and *4EBP* at 2 day post *dsIRS* treatment. (F) The endogenous insulin titers were detected by ELISA. (G-H) Effects of dsRNA treatment on miRNAs (G) and target genes (H) transcript levels in whole bodies. (I-L) The role of the TOR pathway in the miRNA-target axis. (I) Consumption of yeast-rich foods increases TOR transcription. (J-L) Relative expressions of TOR (J), miRNAs (K), and target genes (L) in the whole bodies at 2 day post *dsTOR* injection. Data are expressed as means ± SEM. *, *P* < 0.05; **, *P* < 0.01; ***, *P* < 0.001 (Student’s t-test).

In insects and other rodents, the regulatory mechanisms of systemic energy metabolic homeostasis are closely related to the evolutionarily conserved insulin signal pathway [46, 47]. Feeding can trigger the activity of the insulin signaling pathway, which in turn initiates a cascade of gene transcription. We next examined whether increasing insulin levels could enhance the miRNA-mediated regulatory network. Under yeast-rich conditions, the microinjection of exogenous insulin decreased the expression of the *InR* and *4EBP* and increased the insulin titer, indicating a significantly enhanced insulin pathway activity [48] (Fig 8A and 8B). In addition, microinjection of insulin increased the expression of miR-275 and miR-305 by 56.6% and 56.6% compared to the HCl control, respectively (Fig 8C), but decreased the expression levels of their target genes *SLC2A1* and *GLIS2* to 80.8% and 57.4% of that of the corresponding HCl control, respectively (Fig 8D). Since insulin achieves signaling by phosphorylating insulin receptor substrates upon binding to the insulin receptor, we then detected changes in the expression of miRNA and target genes following RNAi interference with IRS. Injection of IRS dsRNA significantly reduced IRS transcript levels, increased the expression of the *4EBP* and decreased the insulin titer, implying that knockdown of *IRS* effectively inhibited insulin activity (Fig 8E and 8F). Correspondingly, *IRS* knockdown led to 23.4% and 21.5% reduction of miR-275 and miR-305 expression levels, respectively, whereas *SLC2A1* and *GLIS2* transcript levels were increased by 39.4% and 39.2%, respectively (Fig 8G and 8H). Under yeast-free conditions, microinjection of insulin did not enhance the expression of miR-275 and miR-305, indicating that miRNA transcription was dependent on the stimulation of dietary yeast (S5A and S5B Fig).

Besides that, insects sense the available nutrients mainly through the amino acid-TOR signaling pathways, which are known to participate in adjusting growth rates and metabolic processes [49, 50]. Indeed, the transcript level of *TOR* was significantly up-regulated upon the intake of dietary yeast (Fig 8I). To further determine whether *TOR* activity was also required for the miRNA-target axis in *B*. *dorsalis*, we investigated the effect of *TOR* inhibition on the expression levels of the miRNAs and their major target genes. Surprisingly, knockdown of *TOR* exclusively increased the expression of target genes without affecting miRNAs (*SLC2A1*: *P* = 0.0084; *GLIS2*: *P* = 0.0241) (Fig 8J–8L). Taken together, these results suggest that the miR-275/305 cluster is controlled by the insulin signaling pathway rather than the TOR signaling pathway, but that target gene expression is regulated by both pathways.

## Discussion

Dietary yeast comprises a variety of macro- and micronutrients in addition to protein. In many tephritid flies, ingestion of yeast rather than sucrose has been reported to profoundly affect tissue metabolism, lifespan, and reproductive activity [4, 51]. Transcriptomic analysis has revealed that different dietary treatments can affect a variety of metabolic processes, which is conducive to our understanding of complex nutritional regulatory networks at the transcriptional level [23, 24, 45]. Interestingly, miRNAs have emerged as important regulators in modulating larval development, metamorphosis, and reproduction, even as an early embryonic male sex-determining factor [19, 52–54]. However, the relationship between miRNA regulatory cascade and energy metabolism remains poorly understood. The current study provides molecular and genetic support for the conclusion that the miR-275/305 cluster is essential for maintaining energy metabolic homeostasis in *B*. *dorsalis*. Moreover, our data showed that miR-275 and miR-305 acted on the 3’UTR of *SLC2A1* and *GLIS2* to inhibit their transcription, respectively, and knockdown of their target genes partially rescued the metabolic phenotype, suggesting that miRNA-mediated target fine-tuning was essential for the metabolic homeostasis. In addition, we revealed that the insulin signaling pathway is the master regulator of this miRNA-target regulatory network.

miRNA clusters are characterized by their proximity to the same chromosome and co-transcribed by the same promoter in the form of polycistrons, which makes it possible for miRNAs to regulate multiple processes by targeting specific mRNAs [55, 56]. As an evolutionarily conserved miRNA cluster, the miR-275/305 cluster has been initially identified in different insect species, including *D*. *melanogaster* [57], *B*. *mori* [58, 59], *A*. *aegypti* [28, 60, 61] and *B*. *dorsalis* [32, 62].

Our study found that yeast consumption can significantly induce the miR-275/305 cluster’s expression. Similarly, in *A*. *aegypti*, feeding on blood can increase the expression of miR-275 in the fat body and gut, and gut-specific knockdown of the miR-275 prevents blood meal digestion and results in ovarian development defects [33, 63]. Studies in *Drosophila* have also found that feeding can promote miR-305 transcription in the fat body and maintain metabolic adaptation to nutrient deprivation through target dp53 [64]. Foronda et al. found that miR-305 coordinates insulin and Notch pathway activities to maintain the adaptive homeostasis of intestinal epithelial cells [57]. In addition, some studies have shown that miR-305 is involved in the ageing process of *Drosophila* [65]. Our spatial and temporal expression pattern analysis of the miR-275/305 cluster revealed that this cluster was highly expressed in fat-enriched tissues (such as fat body and gut). The primary function of fat is to act as a storage medium for the energy necessary for appropriate growth, development, and reproduction [35, 46]. Based on this, we speculated that the role of the miR-275/305 cluster might be associated with regulating energy metabolism.

Using the CRISPR-Cas9 system, we successfully generated the miR-275- and miR-305-depleted mutants in *B*. *dorsalis*. Our data showed that the mutations affected the structure of the miRNA precursor, thus influencing the processing biogenesis of mature miRNA. Loss of miR-275 and miR-305 activity resulted in seeming phenotypical similarity to wild type, but actual energy metabolic disturbances. The metabolic phenotypes of mutants were similar to those observed in the dilp1–dilp5 deletion flies, suggesting that these might be due to the inability to release insulin-like peptides [66]. TAG is the primary energy substance in the insect fat body, and the increase or decrease of its content will lead to a series of metabolic problems [46, 67]. Flight is one of the most energy-consuming processes in winged insects [35], and the lowered flight capacity could reflect the typical inadequate energy supply. Combining flight observation with metabolic phenotype detection, we speculated that these behavioural phenotype changes might be attributed to aberrant metabolism in mutants, especially the reduction in energy storage substances such as TAG and glycogen. The relationship between metabolism and behaviour has been widely studied. For example, the consumption of high-calorie diets can cause metabolic problems such as obesity, accompanied by an endurance reduction in a repetitive climbing assay [68]. The starved wild-type flies displayed prolonged hyperactivity prior to death due to lack of nutrients available [69]. Ablation of the fly AKH-producing cells significantly reduced trehalose levels, and the fly exhibited progressive hypoactivity [70]. All these findings indicate that metabolic abnormalities can cause phenotypic defects in behaviour. Notably, the significantly increased expression of this cluster was also observed in the larval and pupal stages. These severe metabolic phenotypes observed in the adult mutations may contribute to genetic manipulation altering miRNA expression in these stages, whereas antagomiR injection in adult flies results in relatively mild metabolic readout. However, we cannot conduct specific phenotypic analysis in the early developmental stages, since the mutation is semi-lethal, and distinguishing heterozygotes and wild-type flies is also accompanied by the loss of samples.

Using dual luciferase assay, immunoprecipitation, fluorescence in situ hybridization and bioinformatics analysis, our study demonstrated that miR-275 and miR-305 could act on the 3’UTR of *SLC2A1* and *GLIS2* to inhibit their transcription, respectively. Functional annotation revealed that target genes were involved in several critical physiological processes, such as carbohydrate degradation and fat metabolism. *SLC2A1*, also known as protein glucose transporter 1 (*GLUT1*), is highly conserved across different species and plays a critical role in cellular metabolic processes, including carbohydrate degradation [71, 72]. In murine cells, the transcription level of *GLUT1* has been reported to be associated with cellular response to glucose starvation, indicating that *GLUT1* is negatively regulated by glucose [73–75]. In our miR-275-deleted mutant, the expression level of *SLC2A1* was significantly increased, which might be due to the possibility that mimicking starvation phenotype accelerated glucose intake [35]. On the contrary, knockdown of *SLC2A1* probably blocked the process of carbohydrate degradation, accompanied by an increase in the content of energy storage substances. In *Anopheles stephensi*, silencing *Asteglut1* can significantly elevate the glucose level, thus facilitating *Plasmodium berghei* infection [76]. Similarly, knockdown of *CG7882* belonging to the *SLC2* family increased the circulating glucose levels in *Drosophila* third-instar larvae [45]. The transcription factor Zinc finger protein *GLIS2* homolog, also known as *sugarbabe*, was first identified from *Drosophila*, and the expression of *GLIS2* is strongly induced by a sugar diet [24, 45]. Gene *sugarbabe* can regulate insulin gene expression to manage resource mobilization [52]. In this study, *SLC2A1/GLIS2* knockdown by RNAi successfully rescued the lipid metabolic phenotypes in antagomiR-treated samples. It is worth noting that decreasing the expression of both miRNA and their target genes resulted in reduced glycogen content and increased glucose content. Theoretically, flies with target genes knocked down would exhibit an opposite metabolic phenotype to miRNA mutants. However, our study results showed that miRNA mutants and flies with their corresponding target genes knocked down displayed similar metabolic phenotypes, except for TAG content. One reason for such similar metabolic phenotypes might lie in the direct or indirect regulation of glycogen and glucose metabolism by altering the expression of target genes. Another possible explanation could be that other unidentified target genes might also be involved in this regulation process. Therefore, the rescue of these phenotypes can only be reflected on the TAG level, and the discrepancy between theoretical inference and actual observation remains to be explored. The upcoming challenge is to discover novel target genes involved in energy metabolic homeostasis. Since the interaction relationship between miRNA and their target genes is not conservative across different species, the target genes identified in prior studies are not within the investigation range.

The involvement of the insulin signaling pathway in the regulation of metabolic homeostasis has been well-studied from arthropods to mammals [77, 78]. Therefore, we asked whether this dietary yeast-induced miRNA transcription occurs via the insulin pathway. Our results indicated that exogenous bovine insulin administration significantly increased the transcription of the miRNAs, but suppressed the expression of target genes, indicating that the enhanced insulin activity contributed to the miRNA-mediated regulatory network. We speculated that there might exist binding sites of insulin-responsive genes in the miRNA promoter region to initiate miRNA transcription. In *B*. *mori*, 20-hydroxyecdysone (20E) can synergistically regulate the transcription of the miR-275/305 cluster with its downstream response genes *EcR-B* and *E75B* [79]. Coincidentally, another study reported a similar promoting effect of the 20E signaling pathway on the miR-275/305 cluster in female *A*. *aegypti* mosquitoes [80]. The insulin pathway has been conclusively demonstrated to be involved in controlling the biosynthesis of juvenile hormone (JH) [81–83] and 20-hydroxyecdysone (20E) [84, 85]. Therefore, we speculated that the insulin pathway might regulate the synthesis of 20E, ultimately affecting miRNA transcription. However, more refined experiments are needed to explore the complex regulatory network by insulin-like signaling pathway in *B*. *dorsalis*. In addition, the ingested nutrients are initially sensed via the TOR pathway, which then elicits cascade responses to adjust the metabolic response of the organism. However, in the current study, interfering with the TOR pathway did not affect the transcription level of miRNA, but enhanced the transcription of target genes. Additionally, dietary yeast (mainly containing amino acid) stimulation is essential for the regulation of miRNA transcription. Similarly, in *A*. *coluzzii*, the transcription of miR-276 has been reported to be synergistically regulated by amino acid and 20E after blood ingestion [20]. However, the reason for the amino acid requirement remains to be explored. The above findings indicate that the maintenance of metabolic homeostasis involves the coordination of multiple pathways, and the miRNA pathway is only a part of this regulatory network.

**Fig 9:**
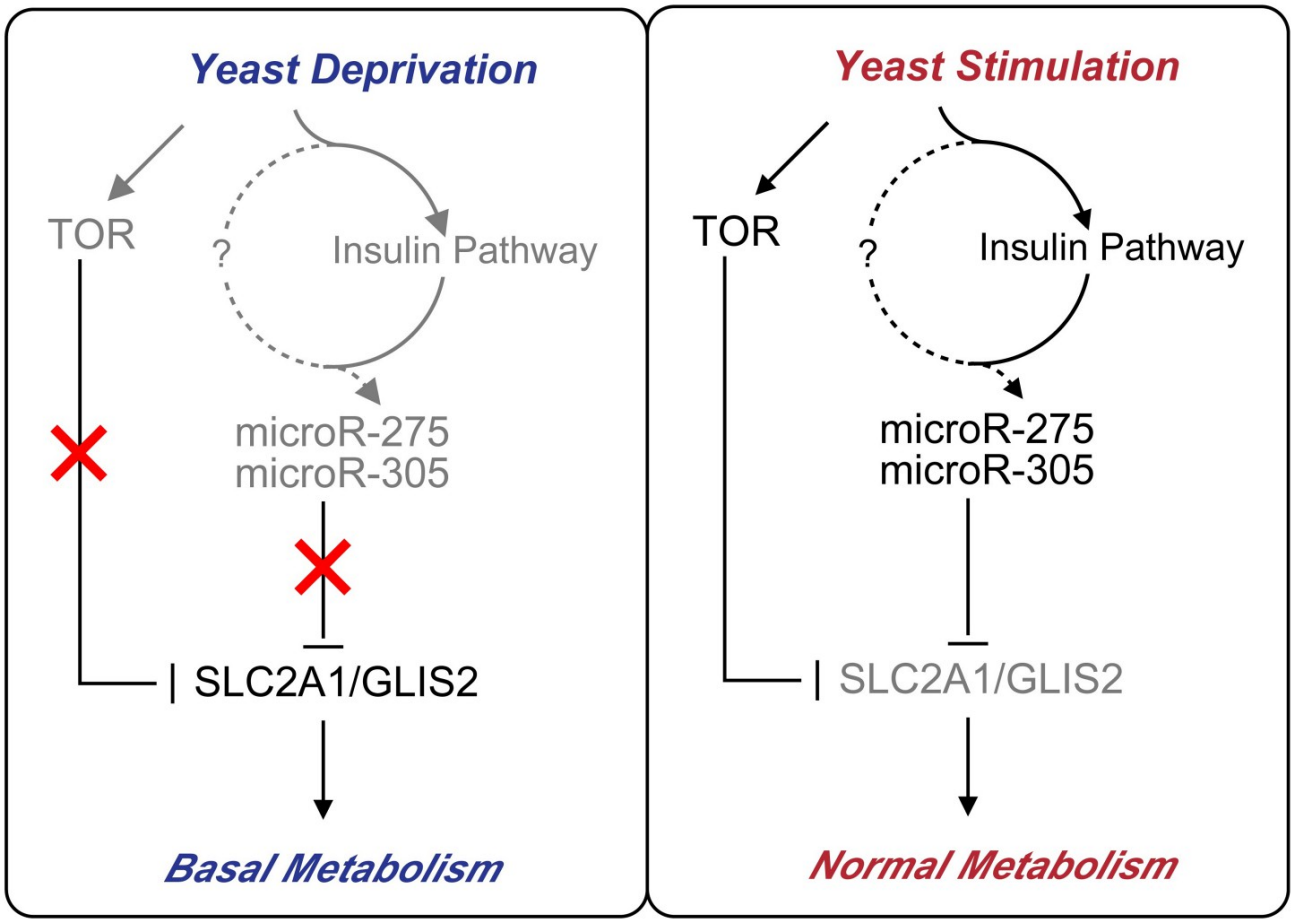
Schematic diagram of miR-275/305 cluster role in modulating dietary yeast-dependent metabolic homeostasis. Our proposed model summarizes the mechanism by which miR-275/305 targets *SLC2A1/GLIS2* to regulate energy metabolic homeostasis. In the presence of dietary yeast, the activation of the insulin signaling pathway is followed by the activation of the miR-275/305 cluster expression, which in turn inhibits *SLC2A1/GLIS2* transcription via binding their 3’UTR region. Meanwhile, TOR can directly act on target genes without affecting miRNAs. This cascade regulation finally ensures normal metabolic physiology. Dietary yeast may contribute to the activation of the miR-275/305 cluster through an unknown pathway (indicated by dashed lines and question marks). Under yeast deprivation conditions, either the activity of insulin or the TOR signaling pathway was low, and the expression of SLC2A1/GLIS2 was derepressed, thus ensuring the basal metabolism needed. Arrows and T-shaped symbols represent activation and inhibition, respectively. Dashed arrows denote the unknown pathway, the components that are less active or inactive are shown in grey, while red crosses indicate shut-down pathways.

In summary, we demonstrated that the miR-275/305 cluster regulated energy metabolic homeostasis by fine-tuning the expression of *SLC2A1* and *GLIS2*, thereby controlling the required energy to ensure normal metabolic physiology in *B*. *dorsalis*. Based on our data, we proposed a transcription regulatory network model for Insulin-miRNA-Target genes in *B*. *dorsalis* (Fig 9). After ingestion of dietary yeast, the activated insulin signaling pathway regulates the transcription of the miR-275/305 cluster, which subsequently binds to the target genes *SLC2A1/GLIS2* to inhibit their transcription at the post-transcriptional level, thus guaranteeing normal energy metabolism. *TOR* also simultaneously regulates target genes without affecting miRNA expression. Dietary yeast may contribute to the activation of the miR-275/305 cluster through an unknown pathway (indicated by dashed lines and question marks). Under yeast deprivation conditions, the activity of TOR and insulin pathways was suppressed, resulting in diminished miRNA-mediated inhibition, manifested by elevated *SLC2A1/GLIS2* expression to maintain the supply of essential metabolic energy. The metabolic homeostasis in response to dietary yeast stimulation is primarily caused by miRNA fune-tune *SLC2A1/GLIS2* expression and can thus be completely reversed by dietary yeast deprivation. Our studies are providing new insights into the regulatory circuitry that controls both miRNA regulation and metabolism output in response to nutrient availability. These findings not only deepen our understanding of energy metabolism homeostasis regulation from miRNAs’ perspective, but also provide a reference for the development of future pest management strategies against *B*. *dorsalis*.

## Materials and methods

### Insect rearing and yeast-refeeding experiments

All flies were reared in the laboratory at 27°C with a relative humidity of 60–70%. After eclosion, flies were separated by sex and fed on an artificial mixture of sucrose and yeast (3:1 w/w) unless otherwise stated.

For the expression pattern experiment, the adult samples were collected from females at regular intervals with the exception of eggs, larvae and pupae. The tissues were sampled from the adult female at 5 day post eclosion (DPE), including the head, thorax, fat body, ovary, gut and malpighian tubules. For yeast re-feeding assays, emerged female flies were randomly divided into three groups (n = 40). Group I and group II were provided with a yeast-free diet (yeast-) and a yeast-rich diet (yeast+) for 6 days, respectively, Group III was fed with a yeast-free diet for 4 days, followed by feeding with the yeast-rich diet for 2 days (refed), in addition to water. All flies were sacrificed to extract RNA on day 6 and stored at -80°C until further processing.

### RNA sequence and miRNA target prediction

Samples from two different dietary treatments (yeast- and yeast+) for 4 days were subjected to small RNA sequencing (HiSeq 4000 single end, 50 bp) to identify miRNA involved in energy metabolism. After removing the adapter dimers, junk, low-complexity sequences, and common RNA families (rRNA, tRNA, snRNA, snoRNA), the unique sequences with a length of 18~26 nt were mapped to specific species precursors using the miRBase 22.0 via BLAST search to identify known miRNAs and novel 3p- and 5p-derived miRNAs. The raw sequencing data were uploaded to the NCBI database (Accession: PRJNA657462). The quantile normalization of miRNA sequencing data was performed according to the previously reported methods [86]. In addition, mRNA sequencing from the same samples was conducted to construct miRNA–mRNA association network. The process is summarized as follows: first, the differentially expressed genes (DEGs) and miRNAs (DEMs) were retained by screening for *P* < *0*.*05* and *FDR* < *0*.*05*. Then, three bioinformatics software (miRanda [38], PITA [37], and TargetScan [36]) were employed to predict the target genes of DEMs. All prediction algorithms were run under default parameters. Lastly, the targets predicted by the significantly up-regulated miRNAs were intersected with the significantly down-regulated mRNAs to identify inversely correlated miRNA-mRNA pairs and *vice versa*.

### Injection of antagomiR, agomiR, dsRNA, and insulin

Chemically modified agomiR and antagomiR were used to increase or inhibit the expression of miR-275 and miR-305. After eclosion, 2-day-old flies were anesthetized on the ice and microinjected with 50 pmol antagomiR or agomiR per fly (50 μM in a volume of 1 μl) or 1 ul dsRNA (2 ug/μl) using a PV820 microinjector (WPI, USA) into the abdomen. Equivalent volumes of antagomiR-NC or agomiR-NC (scrambled sequences), and *dsEGFP* served as negative controls. In rescue experiments, flies were co-injected with the mixture of antagomiR and dsRNA (final concentration of 50 μM antagomiR and 2 μg/μl dsRNA) in the same manner as described above. The miRNA antagomiR or agomiR and their respective controls were purchased from GenePharma (Shanghai, China), and dsRNA was synthesized and purified using the T7 RiboMAX Express RNAi Kit (Promega, USA) following the manufacturer’s protocols. The primer sequences were presented in S3 Table. All experiments were performed with at least five replicates.

To simulate the elevated insulin level, the bovine insulin (5 mg/ml, 1 μl/fly, Sigma) was injected into female adults at 2 DPE, and the same volume of solvent (HCl, PH = 2.0) was used as a control. Total RNA was extracted from flies at 24 h post-injection.

### sgRNA design and embryonic injection

Target sites were designed according to the GG/CN18NGG principle. Templates for synthesizing guide RNA (sgRNA) were generated by annealing two complementary oligonucleotides as our previous description [87]. The PCR program was as follows: denaturation at 98°C for 2 min, followed by 30 cycles of denaturation at 98°C for 5 s, annealing at 55°C for 15 s and extension at 72°C for 30 s. The sgRNAs were synthesized in vitro using the MEGAscript T7 Transcription Kit (Ambion) and purified using the MEGAclear Transcription Clean-Up Kit (Ambion) according to the manufacturer’s instructions. The TrueCut Cas9 Protein v2 was purchased from Invitrogen (USA). The sgRNA cleavage assay *in vitro* was performed in a 10 ul reaction system containing 300 ng DNA template, 0.5 μl of Cas9 protein, 1ul of 10 x reaction buffer, and nuclease-free water at 37°C for 1 h, and the cleavage efficiency was evaluated by electrophoresis on an agarose gel. SG275-3 and SG305-11 with cleavage efficiency of more than 93% were used for subsequent microinjection (S6 Fig).

Prior to the micro-injection, sgRNA and Cas9 nuclease (300 ng/ul Cas9 protein plus 400 ng/μl sgRNA) were co-incubated at 37°C for 15 min to form complex. The embryos were collected within 30 min and injected at the posterior pole. The hatched larvae were then transferred to artificial feed and reared to adulthood. The potential off-target sites were evaluated using CasOT [88]. All the oligonucleotides were synthesized by Genescript (Nanjing, China) and were listed in S3 Table.

### Genome DNA extraction and PCR-RFLP assay of miR-275 and miR-305 mutants

We developed a non-lethal identification method of mutants from gene-edited progeny. In brief, single antennae of G4 (generation 4) was homogenized with a plastic pestle in 40 μl of 5% Chelex-100 (Sigma-Aldrich, C7901), incubated with 2 μl of 20 mg/ml Proteinase K at 56°C for 50 min, and followed by inactivation at 98°C for 10 min. After centrifugation, the supernatant was used as templates to amplify fragments flanking the target loci to obtain 400–800 bp amplification products. The obtained products were purified using DNA FragSelect XP Magnetic Beads (SMART Life Sciences, Changzhou, China) and subsequently digested with the Tsp45I and Bsp1286I (NEB) for the miR-275 and miR-305 mutants, respectively (S7A and S7B Fig). Since Cas9 endonuclease destroyed the restriction enzyme recognition sites at the miRNA locus, heterozygote and homozygote were easily identified by PCR product digestion and electrophoresis.

### Metabolic assays and ELISA analysis

Metabolic assays were performed as previously described [64]. Briefly, five flies were homogenized in 1 ml PBST buffer (0.05% Tween 20) and immediately incubated at 70°C for 5 min to inactivate endogenous enzymes. The homogenate was centrifuged for 5 min at 13,000 g to remove fly debris, and the supernatant was used for the subsequent metabolic determination. TAG was quantified using a serum triglyceride determination kit (Jian Cheng Bioengineering Institute, Nanjing, China) according to the manufacturer’s protocol. The glycogen content was determined using the glycogen assay kit (Beijing Boxbio Science & Technology Co., Ltd., Beijing, China;). For quantification of total sugar content (glucose plus trehalose), samples were incubated with trehalase (Sigma-Aldrich, T8778) at 37°C for 2 h, and subsequently determined using the glucose assay kit (Rongsheng Biotech, Shanghai, China). Metabolite measurement data are normalized to the total protein concentrations obtained by the BCA assay (TransGene Biotech, China). The experiments were conducted with at least seven independent biological replicates. The insulin concentrations were determined by using an ELISA kit (Jiangsu Meimian Industrial Co., Ltd., Yancheng, China) according to the manufacturer’s instructions.

### Flight ability test and negative flip assay

Flight tests were conducted using a 24-channel flight mill system (Jiaduo Group, Hebi, Henan, China), which could automatically record the cumulative flight distance, speed, duration, maximum speed, and other parameters [22, 89–91]. On day 10 post eclosion, flies were mildly anesthetized on ice, and then a flight arm with a length of 13 cm and a diameter of 0.4 mm was attached to the tergum using a droplet of superglue (NEWDON, Germany). The flight direction was kept horizontal which was perpendicular to the flight arm. The experiment’s environment was maintained at 25°C and 70% humidity. Each test was conducted for 4 h with at least 50 flies per treatment.

The negative flip assay was performed by the previously reported method with minor modification [92, 93]. In brief, 4–6-day-old flies were transferred into an observation chamber (20 cm length × 20 cm width × 20 cm height) with ventilation holes on both sides for air permeation. A 30 cm× 30 cm transparent glass lid was covered on the top, which allows light to go through. Motion behavior was recorded using a video camera (Sony FDR-AX40), and the average flip duration was quantified using the Format Factory software (5.3.0.0, Pcfreetime.com). All the tests were performed with the G4 of *B*. *dorsalis*.

### Hatching rate and survival analysis

Mutant flies were cultured in a cage for 12 days until sexual maturity, and then males with the same genotype were introduced to mate. The embryos were collected every 2 days. To measure the hatching rate, about 200 embryos were collected and maintained on wet filter paper, and hatched larvae were counted 2 days later.

For the survival rate analysis, the identified mutants were transferred to a feeding cage and provided with a standard adult diet containing sucrose and yeast (at a 3:1 ratio) and water *ad libitum*. The cages were monitored every day, and the number of dead flies was recorded every day for 40 days.

### Nile Red staining, FISH, and confocal imaging

Lipid droplets were observed using Nile Red staining. Nile Red (Sigma, USA) stock solution (5 mg/mL) was prepared in methyl alcohol, and the working solution (5 μg/mL) was obtained by diluting the stock solution at the ratio of 1:1000 before use. The fat bodies were obtained from 5-day-old adults in 1x PBS, fixed in 4% PFA for 30 min, and washed three times with PBST (PBS + 0.1% Tween 20) for 5 min each. Tissues were stained for 1 h with the Nile Red and DAPI staining solution (Coolaber, China). Samples were washed three times with PBS for 5 min each wash.

RNA in situ hybridization (FISH) was performed as previously described [94, 95]. The tissues were pre-processed following the steps described above, blocked with RNA hybridization buffer (Hyb) containing 50% formamide, 5× SSC, 50 μg/ml heparin, yeast tRNA (0.5 mg/ml), 9.2 mM citric acid, and 0.1% Tween 20 for 3 h at 56°C, and washed for three times in PBST (5 min/wash). Samples were hybridized overnight at 56°C with diluted RNA probe (1:500). After washing three times with PBST (10 min/wash), tissue samples were stained with DAPI for 30 min, and transferred to the mounting medium. Cy3-labeled miR-275/305 probes and FAM-labeled SLC2A1/GLIS2 probes were synthesized by Genescript (Nanjing, China). The details of the oligonucleotide sequences are presented in S3 Table. Images were acquired using an inverted confocal laser scanning microscope (Leica TSC SP8) and processed using Adobe Illustrator.

### Cell culture and luciferase report assay

The HEK293T cell line was cultured in DMEM medium (high glucose) with 10% fetal bovine serum in a 5% CO_2_ atmosphere at 37°C. The 3’UTR sequences of targets containing miRNA binding sites were amplified, cloned into psiCHECK-2 vector (Promega), and verified by sequencing. The mutated UTR sequences in the miRNA binding sites were produced by overlap extension PCR. Primers used for vector construction are presented in S3 Table. The cells seeded on 24-well plates were co-transfected with 500 ng reporter plasmid and 100 nM mimic (final concentration) using Lipofectamine 2000 (Invitrogen), and the mimic-NC (scrambled sequences) was used as a negative control. At 48 h post transfection, the luciferase activity was measured using Dual Luciferase reporter assay system (Promega, WI, USA) on Infinite M200 (TECAN, Switzerland). Renilla luciferase expression was normalized to firefly luciferase expression (relative activity). Each experiment was performed with three biological replicates.

### RNA immunoprecipitation assay (RIP)

RIP assay was conducted using the Magna RIP Kit (Millipore) according to the manufacture’s protocol [96]. Briefly, agomiR-treated abdomen tissues were homogenized in ice-cold RIPA lysis buffer (Beyotime, Shanghai, China), and centrifuged at 12,000 g for 10 min at 4°C. The supernatant was incubated at 4°C overnight with 50 μl protein A/G Beads pre-incubated with a monoclonal antibody against *BdAGO1* or normal Rabbit IgG protein (ABclonal Technology). Subsequently, total RNA was extracted from the precipitated RNA-protein complex, and reverse transcribed into cDNA using the PrimeScript 1st Strand cDNA Synthesis Kit (TaKaRa), followed by quantification using qRT-PCR. The relative expression levels of target genes were normalized to the *α-tubulin* and fold enrichment was quantitated relative to IgG control.

### RNA extraction and qRT-PCR

Total RNAs were isolated with RNAiso Plus (TaKaRa). For gene expression determination, 500 ng RNA was reverse transcribed into cDNA using PrimeScript 1st Strand cDNA Synthesis Kit with gDNA Eraser (TaKaRa). For miRNA quantification, the cDNA was synthesized using miRNA-specific stem-loop RT primers. The qRT-PCR was performed with a 10 μl reaction volume containing 1 μl of diluted cDNA, 5 μl of UNICON qPCR SYBR Green Master Mix (Low ROX) (Yeasen, ShangHai), 0.4 μl of 10 mM of primers, and 3.2 μl of RNase free water using QuantStudio 7 Flex Real-Time PCR System (Applied Biosystems). PCR procedures were as follows: 95°C for 5 min, followed by 40 cycles of 95°C for 10 s and 60°C for 30 s. Melting curve analysis was performed at the end to ensure the specificity of each primer pair. The relative expression levels were calculated by the 2^-ΔΔCt^ method, and α-tubulin and U6 were used as the internal controls of mRNA and miRNA, respectively. The primers used for qRT-PCR are listed in S3 Table.

### Statistical analysis

The unpaired two-tailed Student’s t-test or one-way ANOVA were performed using the SPSS 21.0 software (SPSS Inc., Chicago, USA) to determine the statistically significant differences among groups. *P < 0.05, **P < 0.01, ***P < 0.001, and ****P < 0.0001 represented different levels of significant differences. Graphs were drawn using GraphPad Prism (GraphPad Prism 7.04, USA). Heatmaps were plotted by the “pheatmap” R package.

## Supporting information

S1 Fig Different types of mutations in G0-injected embryos. The PAM sequence is highlighted in grey, and the cleavage site is indicated with a black scissor. Dashed lines in the sequence indicate deleted bases, and red letters represent inserted bases. The numbers of inserted and deleted bases are shown on the right (–, deletion; +, insertion).
(TIF)

S2 Fig miR-275/305 depletion results in eggshell formation defects. (A) Morphological comparison of eggs oviposited from heterozygote mutant and wild-type flies. The eggs were visualized by using the Nikon SM745T stereomicroscope (Scale bar: 1 mm.). (B) The proportion of eggs wrapped by eggshell from the heterozygote mutant and wild type. Embryos were classified as morphologically normal or abnormal based on the presence or absence of eggshells. The miR-275 and miR-305 depleted flies showed 59% and 77% normal embryos, respectively, while the control flies exhibited 98% normal embryos.
(TIF)

S3 Fig AntagomiR-treated flies display metabolic defects similar to miR-275/305 mutants. (A) miRNA inhibition efficiency was determined by qRT-PCR at 2 d post antagomiR injection with scrambled sequences as negative controls (Ant-NC). The results indicated that the mature miR-275 and miR-305 levels decreased to 22.9% and 24.0% of the Ant-NC, respectively. (B-D) Energy metabolic substrate contents in antagomiR-treated samples. (B) TAG content. (C) glycogen content. (D) total sugar content (glucose plus trehalose). Metabolic determination results indicated that the contents of TAG and glycogen in antagomiR treatment group were significantly lower than those in the control group (TAG: Ant-275 vs. Ant-NC, *P* = 0.0069; Ant-305 vs. Ant-NC, *P* = 0.0425; glycogen: Ant-275 vs. Ant-NC, *P* = 0.0390; Ant-305 vs. Ant-NC, *P* = 0.0264), while the total sugar content significantly increased (Ant-275 vs. Ant-NC, *P* > 0.99; Ant-305 vs. Ant-NC, *P* = 0.01). The sample data are obtained from seven replicates. Student t-test was performed to determine statistically significant differences. **, *P* < 0.01; *, *P* < 0.05, NS, not significant.
(TIF)

S4 Fig Putative miRNA binding site in 3’UTR of candidate targets by RNAhybrid software. Red denotes 2–7 bases of miRNA seed sequences. mfe, minimal free energy.
(TIF)

S5 Fig Injection of insulin fails to activate miRNA transcription under yeast-free conditions. (A-B) Effects of exogenous insulin on transcription of *InR* (A), miRNAs (B) at 24 h post insulin injection under yeast-free conditions.
(TIF)

S6 Fig sgRNA cleavage activity assays *in vitro*. The 900bp fragment flanking the miRNA genome sequence was incubated with sgRNA and Cas9 protein at 37°C for 1 h, then the digested products were electrophoresed on 2% agarose gel and visualized. Maker is Trans 2K plus II DNA marker. The cleavage efficiency (EFF) was evaluated using the Gel-Pro analyzer software based on the band brightness.
(TIF)

S7 Fig Identification of progeny mutants using restriction fragment length polymorphism analysis. **(A-B)** The DNA fragments containing the miRNA locus were amplified by PCR from genomic DNA using the specific primer pairs. In the presence of endonuclease, the intact band from the wild-type template can be cleaved into truncated fragments, whereas the band from the homozygous template cannot, and the band from the heterozygous template comprises a mixture of wild-type and homozygote. The endonucleases *Tsp45I* and *Bsp1286I* were used for miR-275 (A) and miR-305 (B) cleavage, respectively.
(TIF)

S1 Table Summary of CRISPR/Cas9-mediated mutations at the miR-275 and miR-305 loci. Injected embryos were raised to adulthood (G0) and crossed to wild-type flies, and transgenic G1 flies were mated within the same family to generate G2 flies, and the final homozygous mutation rate was calculated as G2 homozygous fly number divided by the total G2 fly number.
(XLSX)

S2 Table List of putative target genes obtained from miRNA-mRNA association analysis. (XLSX)

S3 Table List of primer pairs used in this experiment. (XLSX)

S1 Movie Phenotypes of the negative flip of miR-275 mutant flies. (MOV)

S2 Movie Phenotypes of the negative flip of miR-305 mutant flies. (MOV)

S3 Movie Phenotypes of the negative flip of wild-type flies. (MOV)
