# Supplementary figures and images for: miR-275/305 cluster is essential for maintaining energy metabolic homeostasis by the insulin signaling pathway in Bactrocera dorsalis
Source: PLoS Genet. 2022 Oct 5;18(10):e1010418. doi: 10.1371/journal.pgen.1010418 (PMC9534453; doi:10.1371/journal.pgen.1010418)

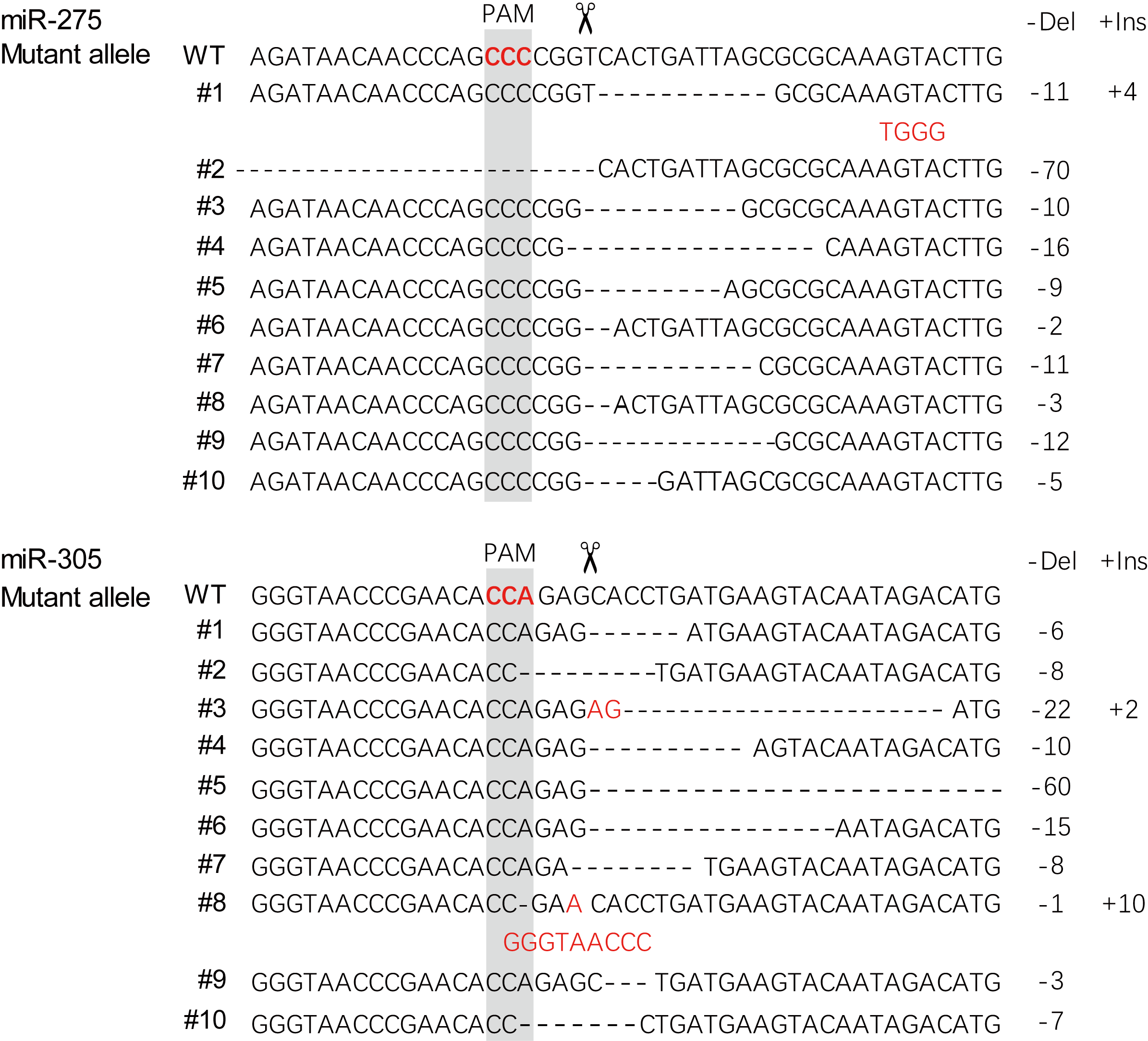

Supplement: S1 Fig — The PAM sequence is highlighted in grey, and the cleavage site is indicated with a black scissor. Dashed lines in the sequence indicate deleted bases, and red letters represent inserted bases. The numbers of inserted and deleted bases are shown on the right (–, deletion; +, insertion). (TIF) [file pgen.1010418.s001.tif]

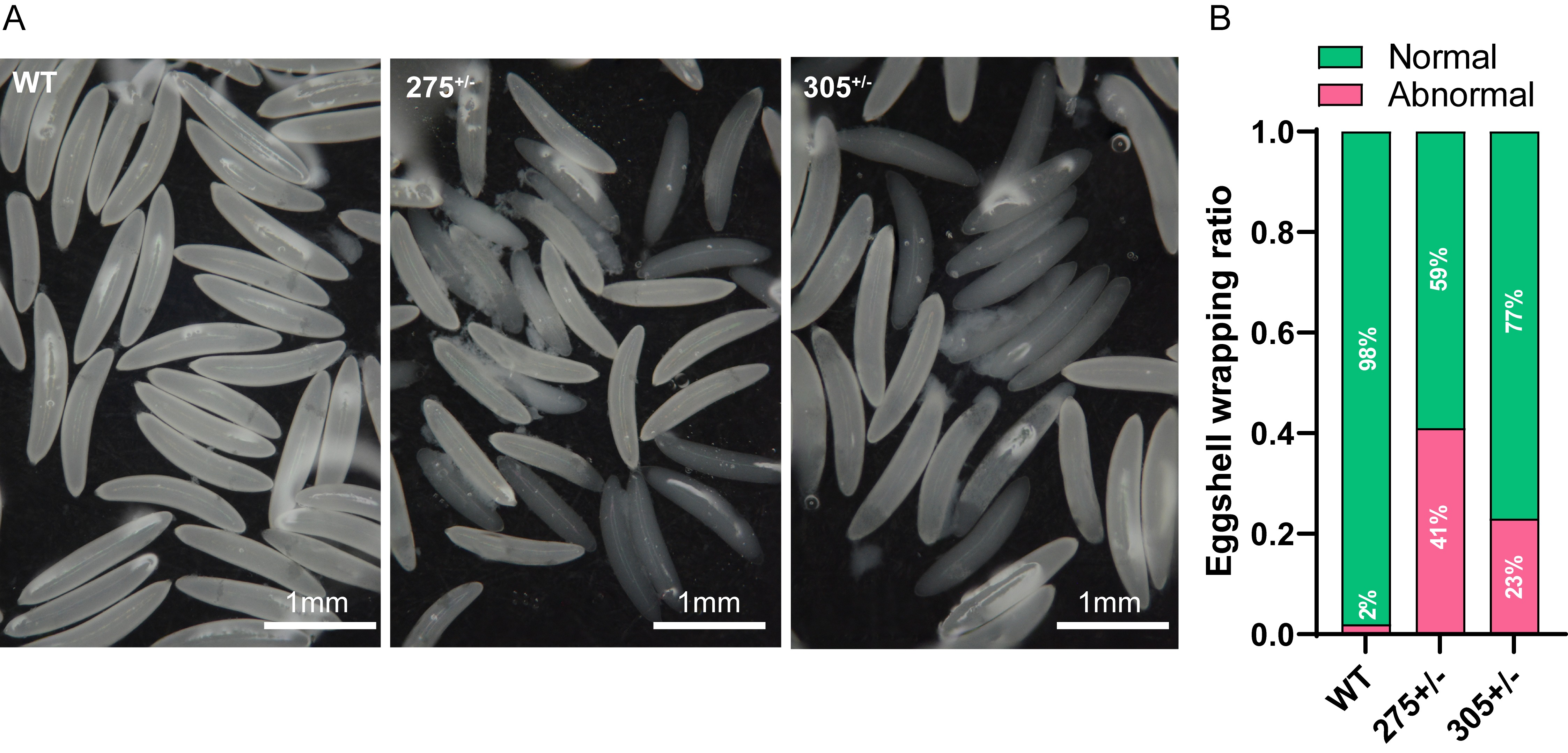

Supplement: S2 Fig — (A) Morphological comparison of eggs oviposited from heterozygote mutant and wild-type flies. The eggs were visualized by using the Nikon SM745T stereomicroscope (Scale bar: 1 mm.). (B) The proportion of eggs wrapped by eggshell from the heterozygote mutant and wild type. Embryos were classified as morphologically normal or abnormal based on the presence or absence of eggshells. The miR-275 and miR-305 depleted flies showed 59% and 77% normal embryos, respectively, while the control flies exhibited 98% normal embryos. (TIF) [file pgen.1010418.s002.tif]

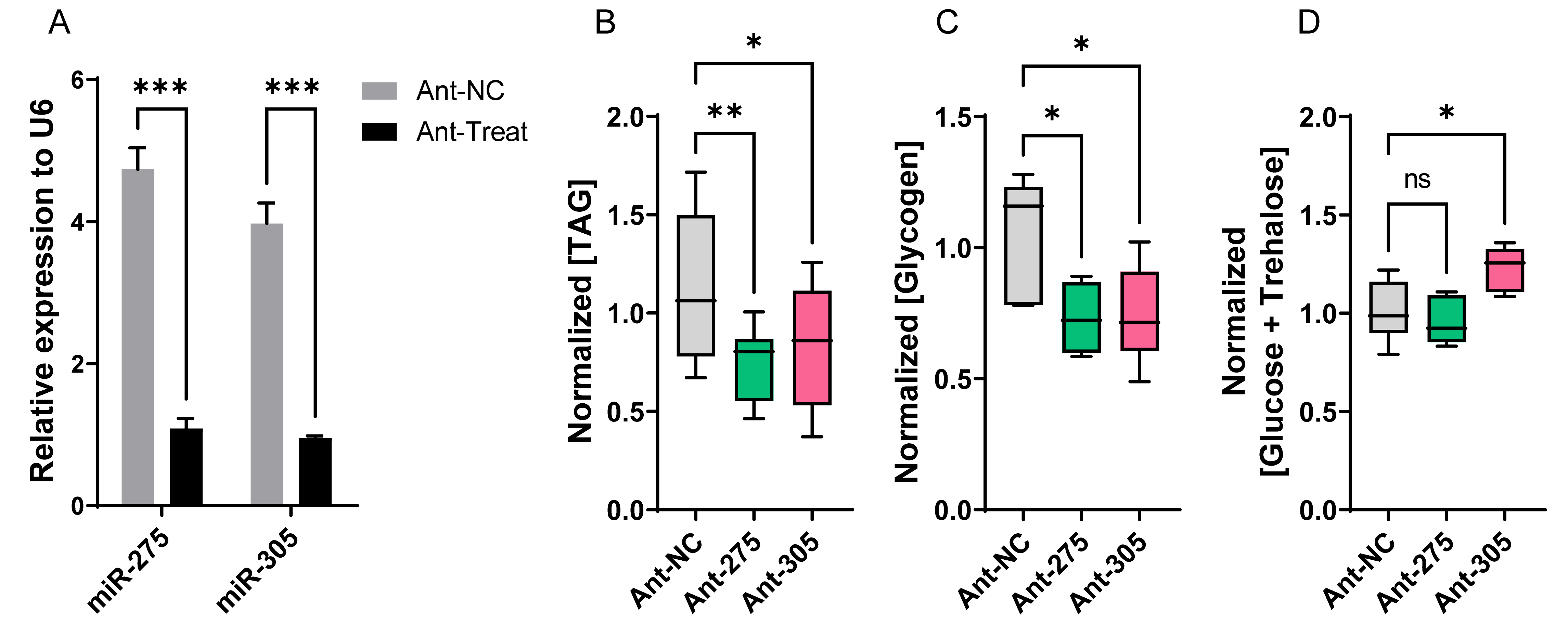

Supplement: S3 Fig — (A) miRNA inhibition efficiency was determined by qRT-PCR at 2 d post antagomiR injection with scrambled sequences as negative controls (Ant-NC). The results indicated that the mature miR-275 and miR-305 levels decreased to 22.9% and 24.0% of the Ant-NC, respectively. (B-D) Energy metabolic substrate contents in antagomiR-treated samples. (B) TAG content. (C) glycogen content. (D) total sugar content (glucose plus trehalose). Metabolic determination results indicated that the contents of TAG and glycogen in antagomiR treatment group were significantly lower than those in the control group (TAG: Ant-275 vs. Ant-NC, P = 0.0069; Ant-305 vs. Ant-NC, P = 0.0425; glycogen: Ant-275 vs. Ant-NC, P = 0.0390; Ant-305 vs. Ant-NC, P = 0.0264), while the total sugar content significantly increased (Ant-275 vs. Ant-NC, P > 0.99; Ant-305 vs. Ant-NC, P = 0.01). The sample data are obtained from seven replicates. Student t-test was performed to determine statistically significant differences. **, P < 0.01; *, P < 0.05, NS, not significant. (TIF) [file pgen.1010418.s003.tif]

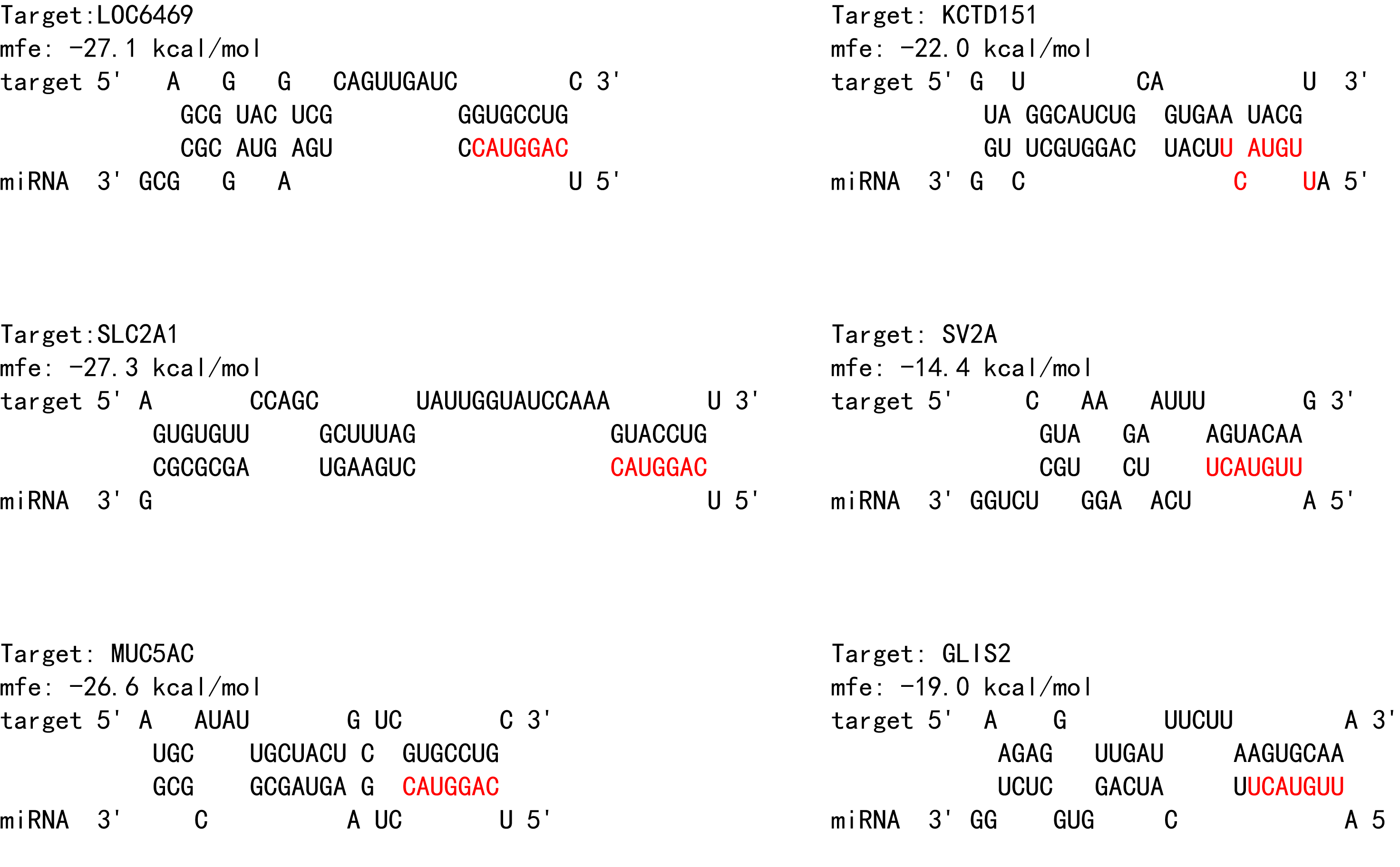

Supplement: S4 Fig — Red denotes 2–7 bases of miRNA seed sequences. mfe, minimal free energy. (TIF) [file pgen.1010418.s004.tif]

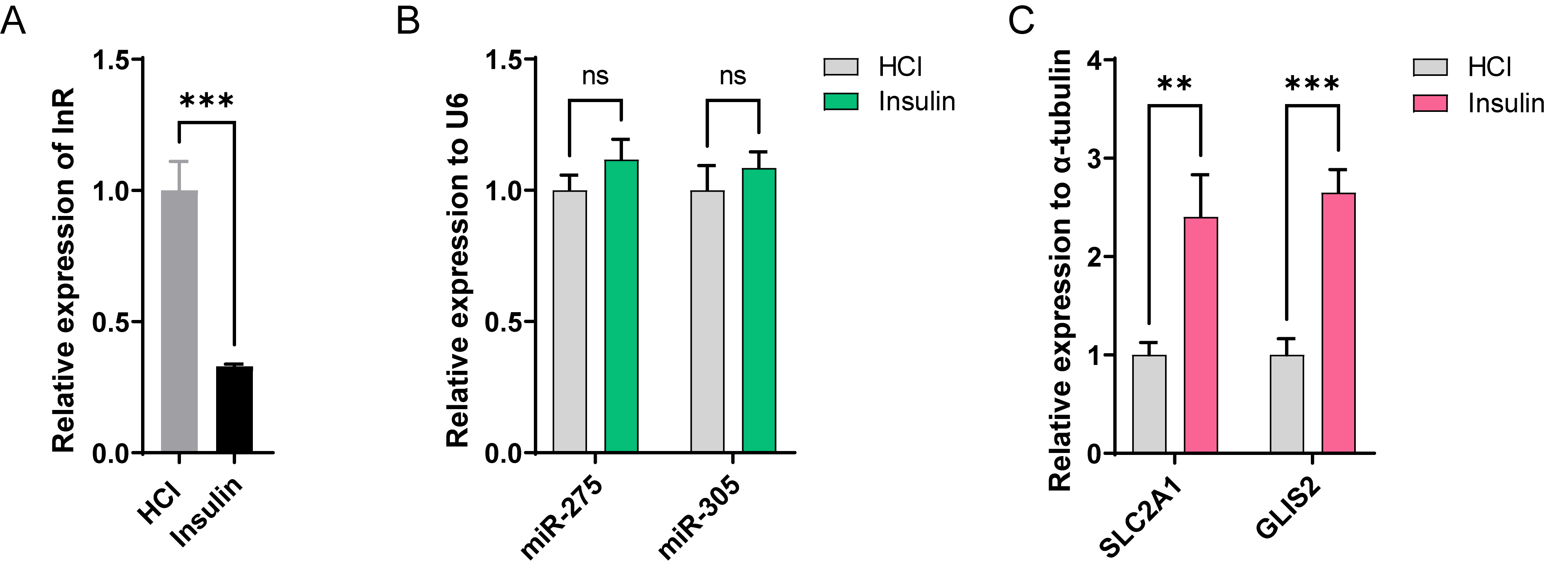

Supplement: S5 Fig — (A-B) Effects of exogenous insulin on transcription of InR (A), miRNAs (B) at 24 h post insulin injection under yeast-free conditions. (TIF) [file pgen.1010418.s005.tif]

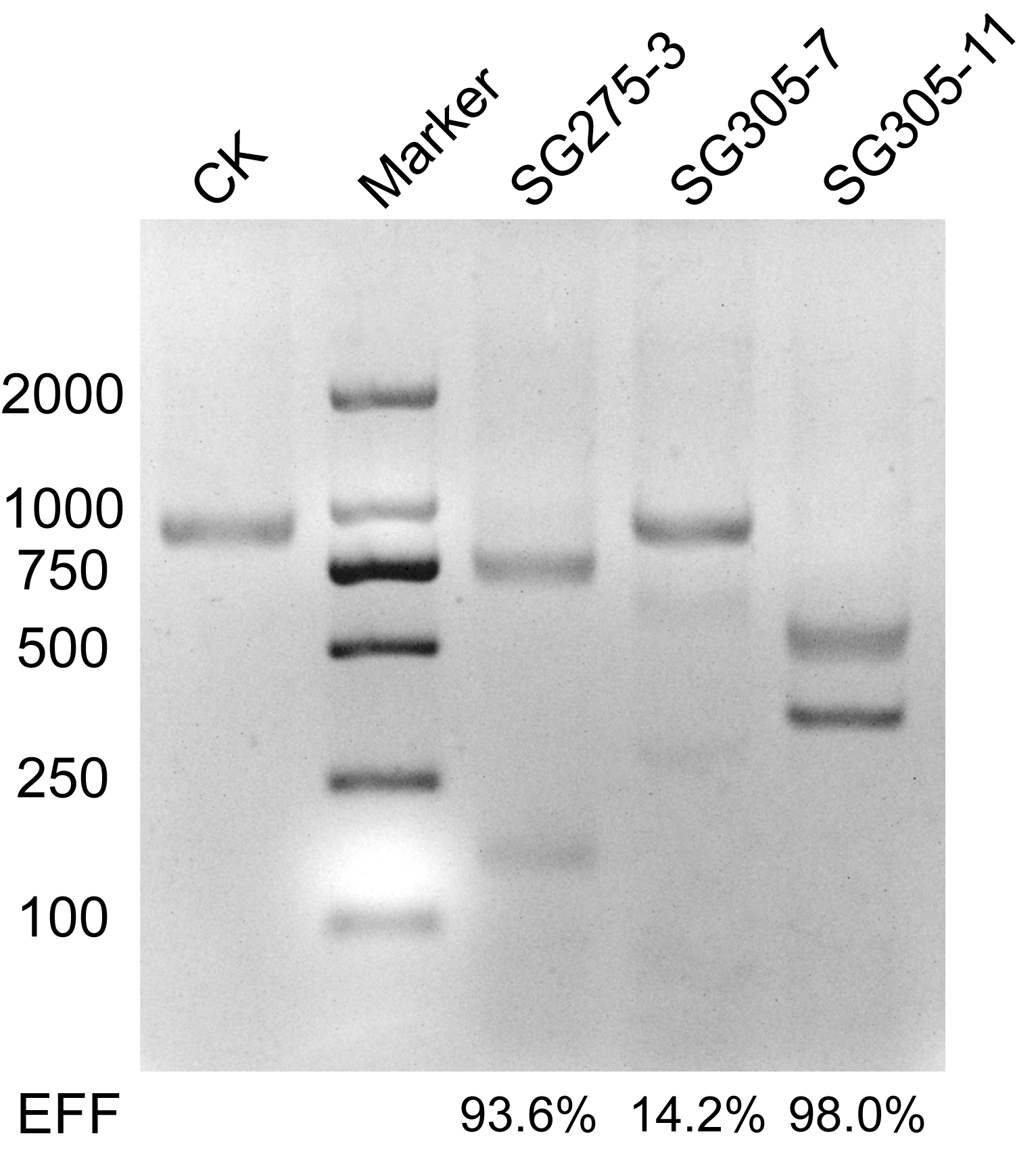

Supplement: S6 Fig — The 900bp fragment flanking the miRNA genome sequence was incubated with sgRNA and Cas9 protein at 37°C for 1 h, then the digested products were electrophoresed on 2% agarose gel and visualized. Maker is Trans 2K plus II DNA marker. The cleavage efficiency (EFF) was evaluated using the Gel-Pro analyzer software based on the band brightness. (TIF) [file pgen.1010418.s006.tif]

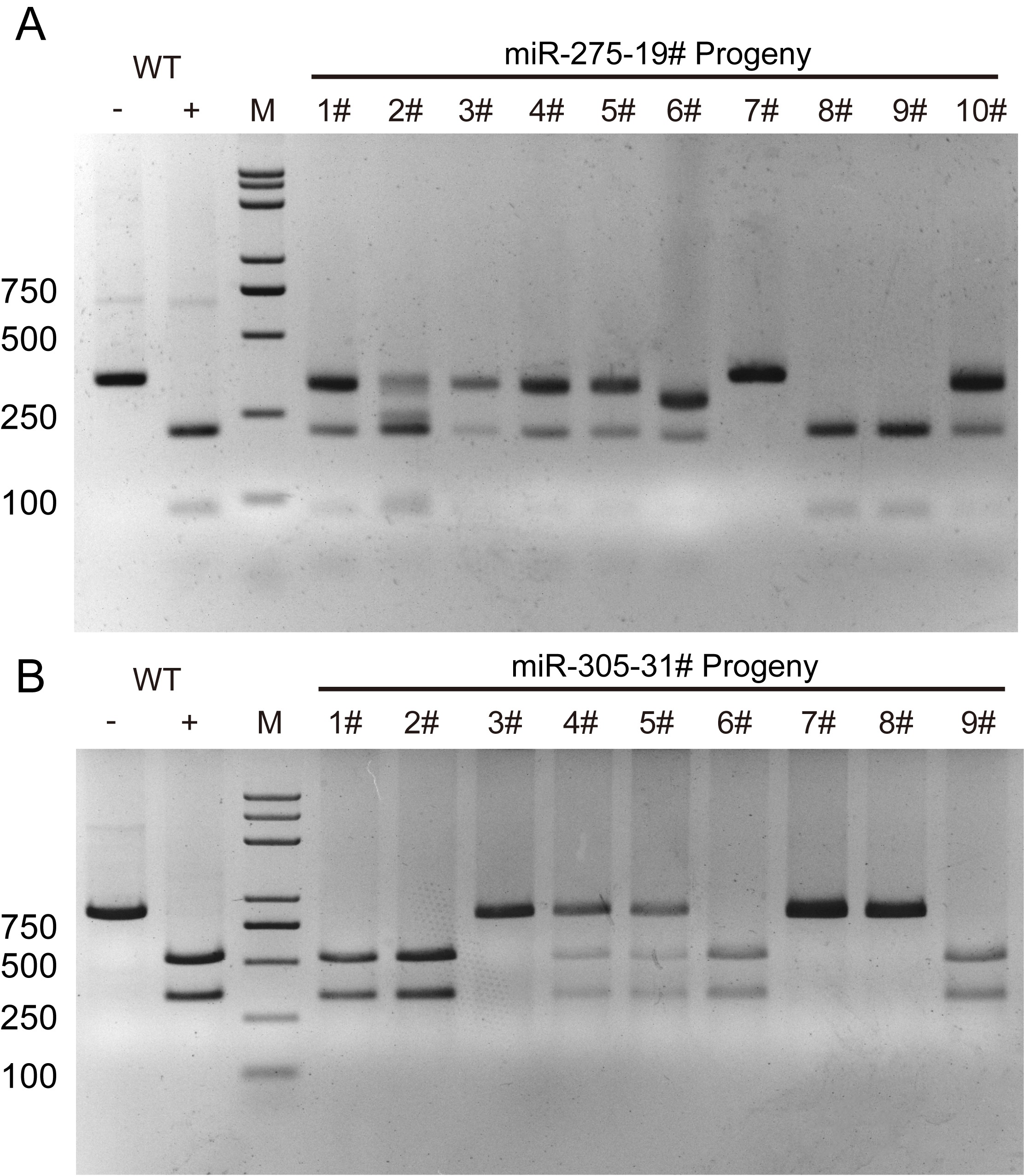

Supplement: S7 Fig — (A-B) The DNA fragments containing the miRNA locus were amplified by PCR from genomic DNA using the specific primer pairs. In the presence of endonuclease, the intact band from the wild-type template can be cleaved into truncated fragments, whereas the band from the homozygous template cannot, and the band from the heterozygous template comprises a mixture of wild-type and homozygote. The endonucleases Tsp45I and Bsp1286I were used for miR-275 (A) and miR-305 (B) cleavage, respectively. (TIF) [file pgen.1010418.s007.tif]
